# A Network Pharmacology Approach to Estimate Potential Targets of the Active Ingredients of Epimedium for Alleviating Mild Cognitive Impairment and Treating Alzheimer's Disease

**DOI:** 10.1155/2021/2302680

**Published:** 2021-01-28

**Authors:** Xianwei Gao, Shengnan Li, Chao Cong, Yuejiao Wang, Lianwei Xu

**Affiliations:** ^1^Longhua Hospital, Shanghai University of Traditional Chinese Medicine, Shanghai 200032, China; ^2^Shanghai University of Traditional Chinese Medicine, Shanghai 201203, China

## Abstract

**Background:**

The present study made use of a network pharmacological approach to evaluate the mechanisms and potential targets of the active ingredients of Epimedium for alleviating mild cognitive impairment (MCI) and treating Alzheimer's disease (AD).

**Methods:**

The active ingredients of Epimedium were acquired from the Traditional Chinese Medicine System Pharmacology database, and potential targets were predicted using the TCMSP target module, SwissTargetPrediction, and PharmMapper database. Target proteins correlating with MCI and AD were downloaded from the GeneCards, DisGeNet, and OMIM databases. The common targets of Epimedium, MCI, and AD were identified using the Jvenn online tool, and a protein-protein interaction (PPI) network was constructed using the String database and Cytoscape. Finally, Gene Ontology (GO) and Kyoto Encyclopedia of Genes and Genomes (KEGG) enrichment analysis of the common targets was performed using DAVID, and molecular docking between active ingredients and target genes was modeled using AutoDock Vina.

**Results:**

A total of 20 active ingredients were analyzed, and 337 compound-related targets were identified for Epimedium. Out of 236 proteins associated with MCI and AD, 54 overlapped with the targets of Epimedium. The top 30 interacting proteins in this set were ranked by topological analysis. GO and KEGG enrichment analysis suggested that the common targets participated in diverse biological processes and pathways, including cell proliferation and apoptosis, inflammatory response, signal transduction, and protein phosphorylation through cancer pathway, MAPK signaling pathway, PI3K-Akt signaling pathway, HIF-1 signaling pathway, sphingolipid signaling pathway, FoxO signaling pathway, and TNF signaling pathway. Molecular docking analysis suggested that the 20 active ingredients could bind to the top 5 protein targets.

**Conclusions:**

The present study provides theoretical evidence for in-depth analysis of the mechanisms and molecular targets by which Epimedium protects against MCI, AD, and other neurodegenerative diseases and lays the foundation for pragmatic clinical applications and potential new drug development.

## 1. Introduction

The incidence of Alzheimer's disease (AD) is increasing significantly with the globally increasing average population age [1]. AD is an irreversible neurodegenerative disease and is the leading cause of dementia [2]. Forty-four million people worldwide currently suffer from dementia, and the number of afflicted people is expected to double by 2050 [3]. AD is thought to occur 20 years or more before symptoms appear, and then progressively deteriorate over time, resulting in the loss of physical function, disability, and death [4]. The pathogenesis of AD is closely related to epigenetic factors, including the environment, vascular disease, inflammation, oxidative stress, or other risk factors, whereas emblematic pathological manifestations include deposition of amyloid-protein (plaque) outside the brain neurons, and tau protein within the brain neurons [5]. These abnormal changes lead to neuronal injury or destruction and a sequence of complicated symptoms. Early symptoms include memory loss, cognitive impairment, apathy, and depression. As the disease progresses, the patient's memory and cognitive impairment gradually worsen, resulting in communication disorders, poor judgment, muddled thoughts, behavioral changes, aphasia, and difficulty swallowing and walking, which drastically interfere with the patient's daily life and impose a burden on the family [6, 7]. The progression and symptoms of AD are continuous and unified, and patients usually transition from normal cognition to mild cognitive impairment (MCI) caused by AD, which further develops into dementia [8]. Epidemiological studies have shown that approximately 10%–15% of people with amnesic-MCI morph into AD; in contrast, only 1% of healthy seniors develop AD [9].

MCI is a clinically recognized neurodegenerative disease that is defined by cognitive changes, and represents a transition stage between healthy aging and dementia [10]. This transition phase is considered to be an optimal treatment window for the protection of brain function, reduction of cognitive impairment, and delay of AD, essentially gaining more time for patients with relatively good cognitive health [11]. Currently, the universal treatments for AD are divided into medication- and non-medication-based therapies. Non-medication therapies can maintain or improve the patient's quality of daily life, but cannot prevent further injury and destruction of neurons. Medication approved by the United States Food and Drug Administration (FDA) for therapeutic use, including rivastigmine, galantamine, donepezil, memantine, and memantine combined with donepezil, can ameliorate the patients' mental state and cognitive symptoms, and slow down memory loss, but the clinical efficacy varies from person to person and the treatment window is limited [12]. MCI is identified as a precursor to AD; however, to date there is no explicit medication for the treatment of MCI and AD. Improving cognitive deficits before the onset of AD can reduce disease severity, and consequently, clinicians often prescribe cholinesterase inhibitors or memantine to alleviate symptoms [13, 14]. Given this situation, the development of new drugs is essential for the effective treatment of MCI, AD, and other neurodegenerative diseases.

Modern pharmacological studies have demonstrated that Epimedium is a flavonoid compound with estrogen-like effects and simultaneously possesses anti-aging, antioxidant, anti-apoptotic, and anti-inflammatory properties [15, 16]. Estrogen protects and regulates mitochondrial function in the brain and plays a significant role in regulating neuroplasticity and synapse formation, with prominent potential for the treatment of AD [17]. These salient effects support the use of Epimedium for the prevention and treatment of various neurodegenerative diseases.

Epimedium, also known as Horny Goat Weed, is a perennial herbaceous plant that is widely applied in Asian countries, such as Japan, South Korea, and China [18, 19]. Epimedium is principally used for curing impotence, infertility, amnesia, osteoporosis, functional diseases of the elderly, cardiovascular diseases, and primary ovarian insufficiency [20]. The Epimedium extract Icariin has been extensively studied in animal models and is able to reduce A*β* production and amyloid plaque deposition, as well as enhancing learning and memory capacity [21]. Icariin mediates the P13K/Akt pathway to inhibit oxidative stress of tau protein, reduce apoptosis, promote cell viability, and prevent oxidative damage *in vitro* [22]. Icariin, which is the dominant active ingredient of Epimedium, exerts protective effects on the nervous system. However, less research has been conducted on the other active ingredients of Epimedium.

Traditional Chinese Medicine (TCM) has the peculiarities of multiple ingredients, multiple targets, and multiple pathways, and using experimental methods to explain the potential mechanisms of a TCM can therefore be difficult. Network pharmacology is a new methodological system based on pharmacology and pharmacodynamics that has the potential to enable exploration of the efficacy of TCMs [23]. By analyzing complicated and multi-layered networks of the active ingredients, targets, and specific therapeutic effects of TCM using molecular biology and correlative databases, the synergistic interaction of multiple compounds and their molecular mechanisms can be elucidated. This method can improve our understanding of TCM and provides a new route for the development of new drugs based on active ingredients [24].

**Figure 1:**
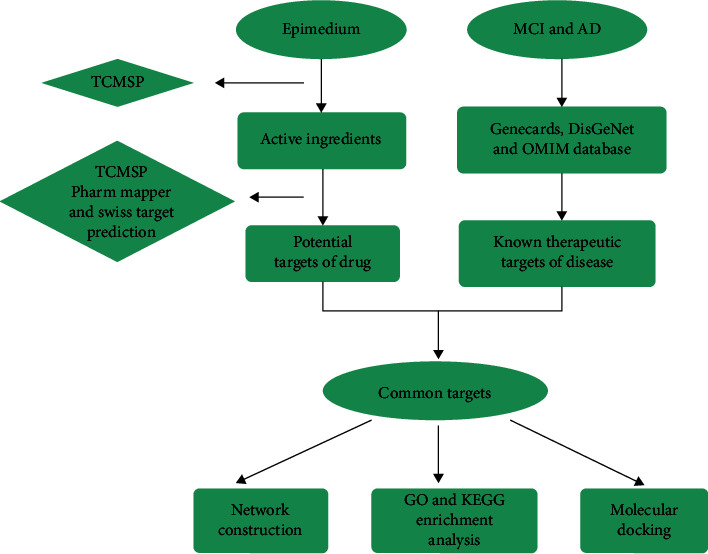
The experimental workflow for Epimedium against MCI and AD.

In the present study, we made use of network pharmacology to investigate the active ingredients, drug targets, and key pathways of Epimedium against MCI and AD. The prediction of interactions between proteins implicated in MCI and AD and the active ingredients of Epimedium will highlight potential targets for further molecular studies, which may uncover the beneficial components of Epimedium and lead to the development of an effective treatment for neurodegenerative diseases. The experimental workflow is shown in Figure 1.

## 2. Materials and Methods

### 2.1. Identification of Active Ingredients in Epimedium

We searched for the active ingredients of Epimedium in the Traditional Chinese Medicine System Pharmacology (TCMSP, http://lsp.nwu.edu.cn/browse.php) database. The TCMSP contains comprehensive information about traditional Chinese herbs, including the chemical structure, oral bioavailability, drug-likeness, half-life, Caco-2 intestinal epithelial permeability, blood-brain barrier, and associated drug-target-disease network. We used pharmacokinetic properties, including absorption, distribution, metabolism and excretion (ADME), oral bioavailability (OB) ≥ 30%, and drug-likeness (DL) ≥ 0. 18%, to screen the active ingredients of Epimedium. Compounds with OB ≥ 30% have good absorption, and a DL ≥ 0. 18% is chemically conducive to drug development [25]. We then obtained the SDF structural formulae of active ingredients from the PubChem database (http://pubchem.ncbi.nlm.nih.gov/) for ulterior target prediction [26].

### 2.2. Target Prediction of Active Ingredients

We used the TCMSP target module, SwissTargetPrediction (http://www.swisstargetprediction.ch/), and PharmMapper (http://www.lilab-ecust.cn/pharmmapper/) databases to predict the potential targets of Epimedium active ingredients. The TCMSP database provides the chemical composition of Chinese herbs, as well as all-sided drug targets of active ingredients, and their relation to diseases [25]. SwissTargetPrediction predicts the targets of active ingredients by comparing their structures to the two- and three-dimensional structures of known compounds [27]. PharmMapper is a freely accessible server that uses pharmacodynamic gene mapping methods to recognize candidate targets for drugs, natural products, or other newly found compounds with unidentified binding targets [28]. The resulting target protein names were ID mapped to their gene names using the UniProt database (http://www.UniProt.org/) [29].

### 2.3. Target Prediction of Disease

GeneCards (https://www.genecards.org/), DisGeNet (http://www.disgenet.org/), and OMIM (https://omim.org/) databases are regularly updated online tools containing information about human genes and genetic diseases. GeneCards includes proteomic, genetic, genomic, transcriptomic, clinical, and other information, which can be applied to predict human gene functions [30]. DisGeNet conveniently integrates the existing databases of mutation sites and human disease-related genes with corresponding information from a mass of literature to construct a unified gene or mutation site disease-related database [31]. OMIM is an authoritative database of human genes and genetic diseases [32]. In the present study, “mild cognitive impairment and Alzheimer's disease” were used as the keywords to collect known therapeutic targets from these three databases for the species “*Homo sapiens*.”

### 2.4. Intersection between Active Ingredients and Disease Targets

We used the Jvenn online tool to generate a Venn diagram and identify common targets for Epimedium, MCI, and AD. Common targets were submitted to String (https://string-db.org/) to generate a protein-protein interaction (PPT) network [33]. The obtained network was visualized using Cytoscape v3. 7. 2, and topological analysis was performed using the network analysis module.

### 2.5. GO/KEGG Pathway Enrichment Analysis

GO is a constantly updated and internationally applicable gene function classification system that can be used to analyze functional enrichment of genes and proteins. KEGG pathway enrichment analysis provided a deeper explanation of different gene and protein functions by identifying enriched biochemical pathways in datasets. To gain insight into the gene functions and signaling pathways of the Epimedium targets, we used DAVID (https://david.ncifcrf.gov/), which is an online biological knowledge base and analysis tool, to identify enriched cellular components (CC), biological processes (BP), molecular function (MF), and KEGG pathways [34].

### 2.6. Validation of Putative Targets by Molecular Docking Analysis

Molecular docking is a burgeoning approach to explore drug mechanisms, elevate the pharmacological effect of drugs to the molecular level, and provide a theoretical basis for treating diseases. In the present study, the top five protein targets of Epimedium were selected based on PPI network analysis and included serum albumin (ALB), RAC-alpha serine/threonine-protein kinase (AKT1), interleukin-6 (IL6), caspase-3 (CASP3), and tumor necrosis factor (TNF), to perform molecular docking with the 20 active ingredients. We retrieved the SDFs of the 20 compounds from Pubchem (https://pubchem.ncbi.nlm.nih.gov/) and used Open Babel 2. 3. 2 software to convert SDFs into PDB files [26]. Next, we used PDB (http://www.rcsb.org/) to obtain the structural data of binding sites on AKT1 (PDB ID: 4EKL), ALB (PDB ID: 6JE7), CASP3 (PDB ID: 2J32), IL6 (PDB ID: 1ALU), and TNF (PDB ID: 5MU8) [35]. We used PyMOL 2. 3. 4 software to perform dehydration and ligand removal on the binding sites. The five proteins were modified using AutoDock Tools for hydrogenation and charge balancing. We used the “grid option” tool to set the grid point spacing to 1, adjust the volume of binding pocket so that the pre-docked molecules can rotate within the box in their most extended state, set the center of pocket as the center of binding site, and convert the protein and ligand small molecules into pdbqt format. Finally, we used AutoDock Vina 1. 1. 2 to perform molecular docking of the five proteins with the 20 ligand small molecules. We selected the receptors and ligands with strong binding free energy and generated three-dimensional graphs in PyMOL to analyze their interactions.

## 3. Results

### 3.1. Active Ingredients of Epimedium

A total of 130 chemical ingredients of Epimedium were obtained from the TCMSP database (Supplementary Table 1). After screening these and confirming their SDF structural formulae in Pubchem, the list was narrowed to 20 active ingredients of Epimedium. The characteristics of the 20 active ingredients are shown in Table 1.

### 3.2. Potential Targets of Active Ingredients

**Figure 2:**
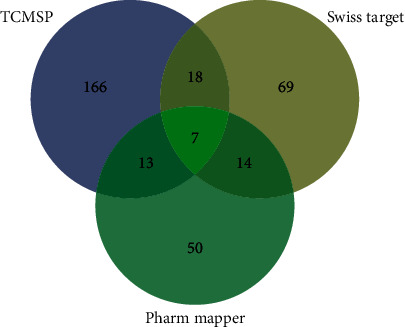
Analysis of potential targets of the active ingredients among TCMSP (blue), SwissTarget (yellow), and PharmMapper (green).

We searched the TCMSP database using the filtered list of Epimedium active ingredients and identified 204 compound-related targets. In addition, the SDF structural formulae of the active ingredients were used to search the SwissTarget and PharmMapper databases, and a list of relevant targets was acquired. The targets were screened to include only those with a probability value ≥0.5 and a Normfit ≥0.8. Using these cutoffs, 108 and 84 compound-related targets were obtained from SwissTarget and PharmMapper, respectively (Supplementary Table 2). After removal of the repeated targets, a total of 337 compound-related targets were identified (Figure 2).

### 3.3. Potential Targets of Disease

The GeneCards, DisGeNet, and OMIM databases were used to identify reviewed therapeutic targets in MCI and AD. The scores in GeneCards and DisGeNet were used to define a set of high-confidence interactions between these diseases and potential targets, with the relevance score ≥20 in GeneCards and score-gda ≥0.1 in DisGeNet. As a result, 546 AD-related targets and 441 MCI-related targets were acquired from GeneCards, and 303 AD-related targets and 5 MCI-related targets were obtained from DisGeNet. In addition, 523 AD-related targets and 85 MCI-related targets were obtained from OMIM. After removing the duplicate targets, 619 potential targets remained for MCI and 1166 for AD, and 236 common targets for both diseases were identified (Supplementary Table 3).

### 3.4. Intersection of Active Ingredients and Disease Targets

**Figure 3:**
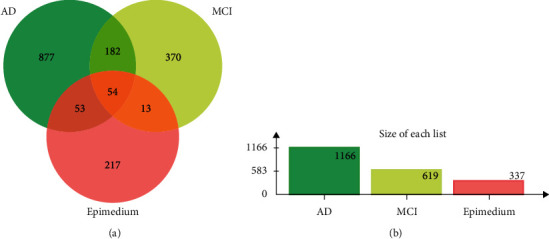
Analysis of predicted active ingredients-disease target genes among Epimedium, MCI, and AD. (a) Numbers of overlapped and specific genes among Epimedium, MCI, and AD. (b) Numbers of predicted genes from each set.

**Figure 4:**
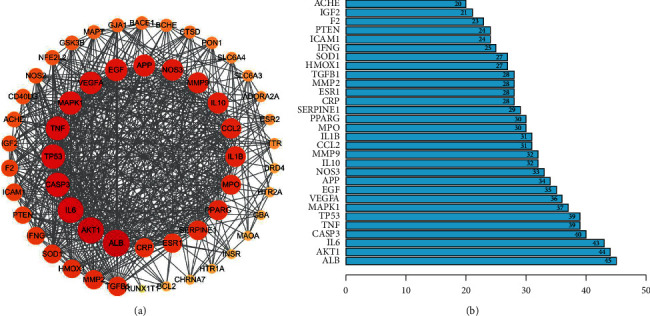
Protein-protein interaction (PPI) network of 54 target genes and top 30 targets from PPI network. (a) Visualization of PPI network of 54 target genes: from orange to red, the larger circle suggests stronger interactions. (b) Top 30 targets from PPI network.

A total of 54 targets overlapped for the Epimedium active ingredients, MCI, and AD. The overlap between the filtered targets was plotted using a Venn diagram (Figure 3). We used String to analyze the interactions between the 54 shared targets. The string analysis contained 54 nodes, 610 link edges, and 195 expected edges at the medium confidence. The average node degree was 22.6, the average local clustering coefficient was 0.737, and the PPI enrichment *P* value was <0.001. Cytoscape software was used to construct the PPI network. Different colors and circle sizes represent different biological information, with larger circles and darker colors (from orange to red) indicating stronger interactions (Figure 4(a)). In addition, we performed a topological analysis of the 54 common targets using a Cytoscape analysis module. The top 30 targets were ranked according to their degree values, represented by a bar graph (Figure 4(b)). Detailed topological analysis results and parameters are presented in Table 2.

### 3.5. GO/KEGG Function and Pathway Enrichment Analysis

**Figure 5:**
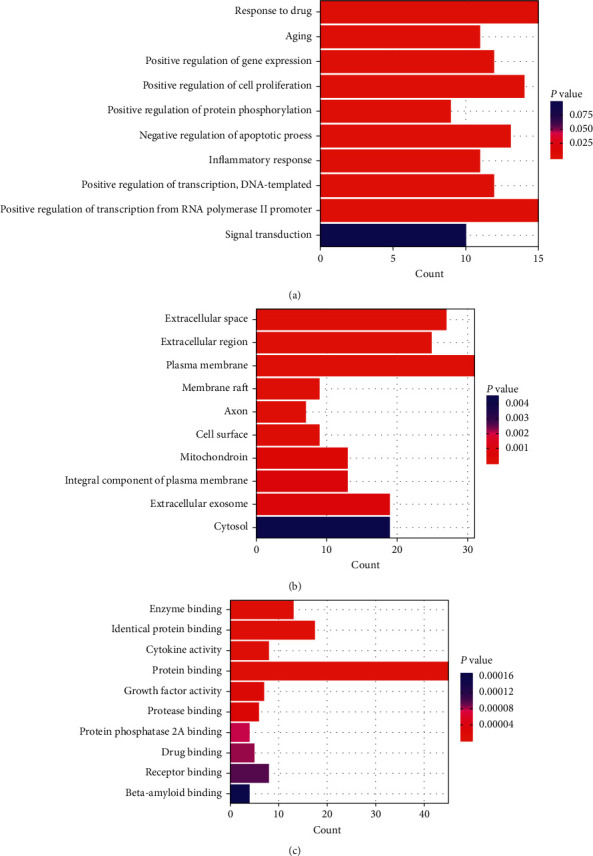
GO enrichment analysis of 54 target genes. (a) Biological process enrichment analysis. (b) Cellular component enrichment analysis. (c) Molecular function enrichment analysis.

To elaborate on the pivotal mechanisms underpinning the neuroprotective activity of Epimedium against MCI and AD, GO enrichment analysis was performed on 54 common targets using DAVID. The GO terms with *P* values <0.01 were considered to be enriched (Supplementary Table 4). In the BP category, regulation of aging was enriched, associated with GO terms related to transcriptional regulation, regulation of cell proliferation and apoptosis, regulation of gene expression, inflammation, signal transduction, and protein phosphorylation (Figure 5(a)). In the CC category, target proteins were primarily distributed in the plasma membrane, and some in the cytosol and mitochondria (Figure 5(b)). In the MF category, target proteins were mainly involved in protein binding, with some involved in beta-amyloid binding (Figure 5(c)).

**Figure 6:**
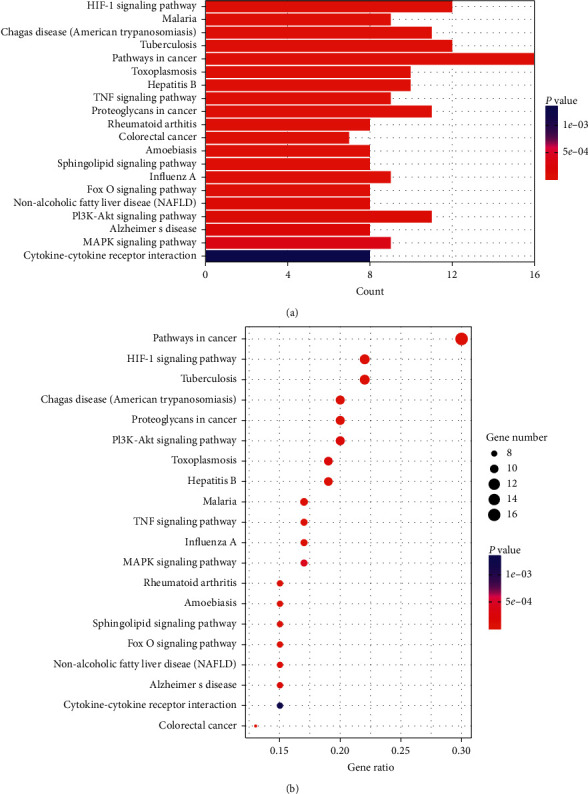
KEGG enrichment analysis of 54 target genes. (a) Barplot of KEGG analysis. (b) Dotplot of KEGG analysis: the size of dots indicates the number of target genes; the different color of dots indicates different *P* value ranges.

To identify pathways that are enriched, and therefore likely to be involved in the neuroprotective effects of Epimedium, we performed KEGG enrichment analysis of the 54 potential targets using DAVID, with a *P* value cutoff of <0.01. We selected the top 20 enriched pathways, which were represented as bar and dot plots (Figure 6).

**Figure 7:**
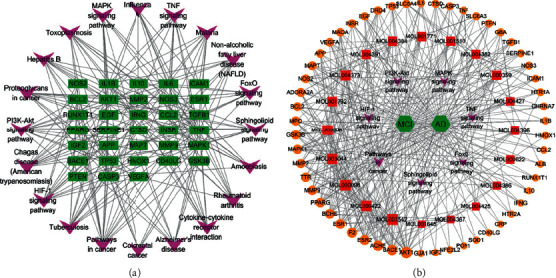
Analysis of targets-pathway network. (a) Network of 20 pathways and their shared targets: pathways were represented by arrowheads (purple), shared targets by rectangle (green). (b) An integral network of Epimedium active ingredients, MCI, and AD: Epimedium active ingredients were represented by square (red), disease by hexagons (green), target genes by circular (orange), and pathways by arrowheads (purple).

In addition, we identified targets that were shared between multiple pathways, which are listed in Supplementary Table 5. We constructed a target-pathway network, as shown in Figure 7(a). To understand the mutual effects of Epimedium, target, disease, and pathway, seven typical pathways were selected to set up an integral network, including the cancer pathway, mitogen-activated protein kinase (MAPK) signaling pathway, PI3K-Akt signaling pathway, HIF-1 signaling pathway, sphingolipid signaling pathway, FoxO signaling pathway, and TNF signaling pathway. Thus, an intricate network was formed to describe the effects of Epimedium active ingredients and potential targets of MCI and AD (Figure 7(b)).

### 3.6. Molecular Docking between Active Ingredients and Potential Targets

**Figure 8:**
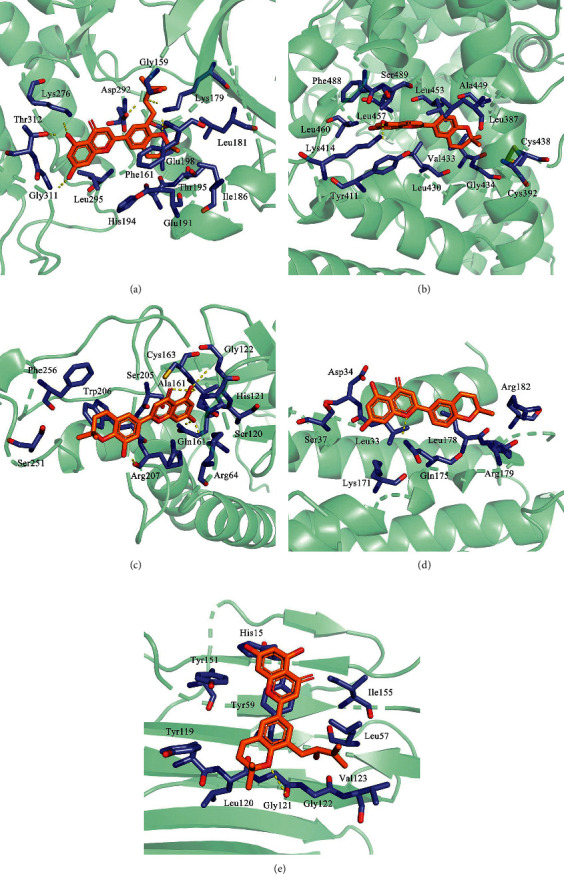
The molecular docking results of active ingredients and targets. (a) Binding pattern between Yinyanghuo A and AKT1. (b) Binding pattern between Yinyanghuo C and ALB. (c) Binding pattern between Yinyanghuo E and CASP3. (d) Binding pattern between Yinyanghuo C and IL6. (e) Binding pattern between Yinyanghuo A and TNF.

To validate the interactions between the screened Epimedium active ingredients and their proposed targets, we conducted molecular docking experiments. The binding free energy score and docking parameters are shown in Table 3. AutoDock Vina outputs the results in the form of an affinity energy, which is assessed by calculating the spatial effect, repulsion, hydrogen bonding, hydrophobic interactions, and molecular flexibility of the receptor-ligand complexes. The affinity energy is a prerequisite indicator to determine whether the ligand small molecule can effectively bind to the receptor, with lower energy values suggesting better binding capacity for the receptor and ligand. According to this scoring system, AKT1 and Yinyanghuo A, ALB and Yinyanghuo C, CASP3 and Yinyanghuo E, IL6 and Yinyanghuo C, and TNF and Yinyanghuo A were selected for further analysis (Figure 8).

## 4. Discussion

In terms of TCM theory, MCI and AD fall under the category of amnesia and dementia. The same treatment for different diseases (Yi Bing Tong Zhi) is a fundamental treatment principle of TCM, which has accumulated vast experience in treating dementia over hundreds of years of clinical practice. Epimedium is one of the cross-sectional herbs that is prescribed as part of Chinese medicine, owing to its anti-apoptotic and anti-oxidative properties, and it is frequently applied to cure central nervous system diseases like forgetfulness, cognitive impairment, and dementia. The mechanism by which Epimedium exerts its therapeutic function is still poorly defined due to the complexity of its constituents. To enable better understanding of the mechanisms behind the protective effects of Epimedium, in the present study we made use of network pharmacology to explore multiple molecular perspectives, and to provide a theoretical basis for the treatment of neurodegenerative diseases.

We identified 20 chemical compounds as the potential active ingredients of Epimedium, many of which have been widely reported to possess inhibitory effects on neurological aging. For instance, icariin was used in Tg2576 mice (containing the Swedish AD mutant gene), with a standard dose of 60 mg/kg. After three months of administration, the hippocampal nerve regeneration and working memory ability of the mice had improved [36]. Icariin also reduced the expression of amyloid *β*1-42 and prevented the expression of amyloid precursor protein and *β*-site APP cleaving enzyme 1 in AD animal models [37]. Another active ingredient, kaempferol, inhibited the oxidative stress response and neuroinflammation in transgenic AD drosophila and an ovariectomized rat model, as well as reversing the loss of climbing ability and improving memory functions [38]. Quercetin is a flavonoid compound that protects neurons from oxidative and excitotoxic effects by modulating cell death mechanisms [39]. It also reduces A*β* polymerization, APP, and BACE1 expression and reduces oxidative stress [40]. Our molecular docking results predicted probable interactions between several active ingredients of Epimedium and protein targets that are related to MCI and AD. These predicted interactions imply that the active ingredients of Epimedium interact with proteins that have known functions in neurological diseases, which may explain its therapeutic effects. Based on these results, Epimedium and its active ingredients should be studied further, with a view to developing novel drugs for the treatment and prevention of MCI, AD, and other neurodegenerative diseases.

In the present study, KEGG pathway and GO enrichment analysis was carried out for 54 common targets of Epimedium, MCI, and AD. Based on these analyses, we identified a subset of proteins, AKT1, MAPK1, CASP3, IL6, TNF, BCL2, TP53 and NOS3, which exert their effects via multiple different biochemical pathways. Such proteins are likely to be “master regulators” and could be major mediators of neurodegenerative diseases. For example, AKT1 regulates cell survival, proliferation, and growth and metabolism mediated by serine and threonine phosphorylation of downstream substrates [41, 42]. AKT positively regulates CREB1 protein activity and plays a crucial role in the regulation of NF-*κ*B-dependent gene transcription. CREB1 phosphorylation induces the binding of auxiliary proteins that are also necessary for the transcription of pro-survival genes, such as BCL2 and MCL1 [43]. The MAPK/ERK cascade accommodates biological functions such as cell survival, growth, adhesion, and differentiation and phosphorylates transcription factors and regulates meiosis and mitosis in differentiated cells [44].

BCL2 is a pivotal apoptosis regulator in the apoptotic signaling pathway. BCL2 inhibits apoptosis in the outer mitochondrial membrane and inhibits the reduction of glutathione and redox state, controls membrane potential, and prevents the release of pro-apoptotic proteins such as cytochrome C and apoptosis-inducing factor AIF to suppress apoptosis [45, 46]. Caspase-3 (CASP3) is an important pro-apoptotic protease that effects apoptosis mediators such as BCL2 and Bax. BCL2 is a direct substrate upstream of CASP3. BCL2 and CASP3 are both interrelated and mutually constrained and are jointly involved in the process of neuronal apoptosis [47, 48]. Interleukin-6 (IL6) is a cellular inflammatory factor secreted by monocytes and is involved in neuroinflammatory processes along with IL10 and IL1*β*. IL6 exacerbates the expression of inflammatory factor IL10 in downstream signaling pathways and promotes the oxidative stress response, leading to apoptosis or atrophy of neuronal cells. Moreover, IL6 promotes *β*-amyloid deposition and affects the integrity of neuronal axons or dendritic membranes [49, 50].

The MAPK signaling pathway is one of the essential signaling systems in living organisms and has been broadly studied in plants, insects, and mammals. MAPK signaling participates in a wide variety of physiopathological processes, such as cell growth, development, differentiation, and apoptosis, and is a regulator of inflammation and the stress response [51]. Neuroinflammation is the subject of recent attention as a latent element in the pathology of AD. Massive *β*-amyloid deposition and hyperphosphorylation of tau protein bring about cell autophagy, function loss, and apoptosis, and these pathological reactions are compactly associated with MAPK activation and signal conduction [52]. Pinocembrin, a natural flavonoid compound, inhibits RAGE expression both *in vivo* and *in vitro*, preventing P38MAPK and MK2 activation and downregulating the NF-*κ*B and SAPK/JNK-c-Jun signaling pathways to reduce cerebral cortex neurodegeneration and ameliorate cognitive function [53, 54]. Drugs that directly target the MAPK signaling pathway may prove to be an effective strategy for the treatment of AD.

The PI3K-Akt signaling pathway participates in the processes of cell reproduction, differentiation, and apoptosis and has many effectors downstream. Akt is the central link in the PI3K-Akt signaling pathway, and it translocates from the cell membrane to the nucleus, where it affects many transcriptional regulators and factors after activation, such as NF-*κ*B, CREB, mTOR, GSK-3*β*, caspase, and the BCL2 family [55]. The PI3K-Akt signaling pathway is involved in the energy metabolism of the aging process, as well as numerous physiological-pathological processes of age-related diseases. Polyphenolic anthocyanins are a natural compound with antioxidant and neuroprotective features that reduce the production of A*β* and ROS in *in vitro* AD models and APP/PS1 transgenic mouse models. Polyphenolic anthocyanins reduce oxidative stress to exert their neuroprotective effects, improve memory dysfunction, and delay neurodegenerative diseases through the regulation of the PI3K/Akt/Nrf2 pathway [56].

The HIF-1 signaling pathway is a physiological response to hypoxia that is mediated by hypoxia-inducible factors (HIFs) and activated or initiated in mammals to adapt to hypoxic environments, also allowing for the accommodation of hypoxia and increased cellular energy metabolism [57]. HIFs are involved in many disease processes, such as inflammation-induced cancer exacerbation, angiogenesis, malignant cell proliferation, and anti-apoptosis [58]. During aging, the supply of oxygen to the brain gradually decreases, and metabolism slows affecting APP expression and transport, leading to an imbalance between the production and clearance of A*β* and increasing the risk of AD [59]. Animal experiments suggest that HIF-1*α* upregulates BACE1 expression and catalyzes *β*-cleavage of APP, while BACE1 expression in the hippocampus and cerebral cortex of mice is reduced by the lack of HIF-1*α*. The above examples demonstrate that the HIF-1 signaling pathway is one of the most important pathways for regulating APP amyloidosis [60].

The sphingolipid signaling pathway plays crucial roles in aging, neurodegenerative diseases, and immune regulation [61]. Sphingosine, sphingosine-1-phosphate, ceramide, and ceramide-1-phosphate are functionally similar and have modulatory effects on proliferation, differentiation, maturation, aging, death, stress response, and inflammation in central nervous cells and are currently the strongest known biologically active sphingomyelin compounds [62]. For example, A*β*25-35 was applied to SH-SY5Y neuronal cells to create an AD model to induce SPHK1 inhibition and neuroamide accumulation, but the upregulation of sphingosine kinase prevented cell death and exerted a protective function [63]. Sphingolipids are pro-secretory molecules that regulate APP secretion and directly protect the nervous system of AD patients, which have excellent prospects for clinical application [64].

Forkhead box class O (FoxO) is a mammalian forkhead transcription factor that interferes with differentiated cell survival and stem cell proliferation and is a potential target for the control of neurological diseases [65]. The FoxO transcription factor interacts with numerous signaling pathways, such as Akt, SIRT1, IKK, and growth factor [66, 67]. FoxO proteins are selectively expressed throughout the body and exert different biological functions in the nervous system [68]. The complex interactions between FoxO proteins and their signal transduction pathways are capable of promoting cellular autophagy, exerting antioxidant effects under oxidative stress, improving cell survival, and scavenging toxic proteins [69]. FoxO proteins hold promise as a new strategy for the treatment of neurodegenerative diseases.

Tumor necrosis factor (TNF) is a cytokine ligand that participates in systemic inflammation. Both TNF and its type 1 receptor (TNFRI) are involved in AD-associated cerebral neuroinflammation and regulate amyloid formation through secreted enzymes [70]. Aging, injury, or degenerative neurological conditions contribute to the production and release of TNF*α*, inducing destructive or toxic biological effects when it binds to specific receptors [71]. Developing drugs to modulate the production of TNF*α*, such as NF-*κ*B, P38 MAPK, and TNF receptor antagonist inhibitors, may be potential candidates for the treatment of cognitive impairment, AD, and other neurodegenerative diseases.

As mentioned above, the targets identified in our study are consistent with previous attempts. Epimedium active ingredients have confirmed interacting partners with proteins that are important regulators of health and disease; these interactions could point future research towards novel therapeutic strategies for treating neurodegenerative diseases.

## 5. Conclusions

In summary, we made use of network pharmacology to reveal the interactions between the possible active ingredients of Epimedium and known therapeutic genes associated with MCI and AD. Through further screening, we verified the potential targets of Epimedium at the molecular level and predicted possible major regulatory pathways that are involved in its neuroprotective effects. The pathogenesis of MCI and AD is complicated, forming a multi-path and multi-target action mode with interlaced signal pathways. The active ingredients of Epimedium against MCI and AD may function by modulating these pathways, demonstrating the multi-component and multi-target capabilities of Chinese herbs. Our findings provide a hypothetical basis for further practical molecular studies of the interactions between Epimedium and these proteins. The combination of Chinese medicine and modern analytical methods will allow in-depth studies of Epimedium's efficacy at treating neurodegenerative diseases. Our findings could lay the foundation for pragmatic clinical application of Epimedium, and new drug development.

## Figures and Tables

**Table 1:** The characteristics of active ingredients in Epimedium.

Mol ID	Molecule name	Molecular formula	OB (%)	DL
MOL001510	24-epicampesterol	C_28_H_48_O	37.58	0.71
MOL001645	Linoleyl acetate	C_20_H_36_O_2_	42.10	0.20
MOL001771	Poriferast-5-en-3beta-ol	C_29_H_50_O	36.91	0.75
MOL001792	DFV	C_15_H_12_O_4_	32.76	0.18
MOL003044	Chryseriol	C_16_H_12_O_6_	35.85	0.27
MOL003542	8-lsopentenyl-kaempferol	C_20_H_18_O_6_	38.04	0.39
MOL000359	Sitosterol	C_29_H_50_O	36.91	0.75
MOL000422	Kaempferol	C_15_H_10_O_6_	41.88	0.24
MOL004367	Olivil	C_20_H_24_O_7_	62.23	0.41
MOL004373	Anhydroicaritin	C_21_H_20_O_6_	45.41	0.44
MOL004382	Yinyanghuo A	C_25_H_24_O_6_	56.96	0.77
MOL004384	Yinyanghuo C	C_20_H_16_O_5_	45.67	0.50
MOL004386	Yinyanghuo E	C_20_H_16_O_6_	51.63	0.55
MOL004391	8-(3-methylbut-2-enyl)-2-phenyl-chromone	C_20_H_18_O_2_	48.54	0.25
MOL004396	1,2-bis(4-hydroxy-3-methoxyphenyl) propane-1,3-diol	C_17_H_20_O_6_	52.31	0.22
MOL004425	Icariin	C_33_H_40_O_15_	41.58	0.61
MOL004427	Icariside A7	C_23_H_26_O_10_	31.91	0.86
MOL000006	Luteolin	C_15_H_10_O_6_	36.16	0.25
MOL000622	Magnograndiolide	C_15_H_22_O_4_	63.71	0.19
MOL000098	Quercetin	C_15_H_10_O_7_	46.43	0.28

**Table 2:** The topological analysis of 54 genes shared by Epimedium, MCI, and AD. Data were ranked by degree.

Target	Name	Degree	Betweenness centrality	Closeness centrality
ALB	Serum albumin	45	0.061	0.869
AKT1	RAC-alpha serine/threonine-protein kinase	44	0.080	0.855
IL6	Interleukin-6	43	0.045	0.841
CASP3	Caspase-3	40	0.025	0.803
TNF	Tumor necrosis factor	39	0.021	0.791
TP53	Cellular tumor antigen p53	39	0.041	0.779
MAPK1	Mitogen-activated protein kinase 1	37	0.039	0.757
VEGFA	Vascular endothelial growth factor A	36	0.015	0.746
EGF	Pro-epidermal growth factor	35	0.020	0.746
APP	Amyloid-beta precursor protein	34	0.069	0.736
NOS3	Nitric oxide synthase, endothelial	33	0.015	0.726
IL10	Interleukin-10	32	0.009	0.707
MMP9	Matrix metalloproteinase-9	32	0.006	0.707
CCL2	C-C motif chemokine 2	31	0.005	0.697
IL1B	Interleukin-1 beta	31	0.010	0.707
MPO	Myeloperoxidase	30	0.009	0.697
PPARG	Peroxisome proliferator-activated receptor gamma	30	0.008	0.688
SERPINE1	Plasminogen activator inhibitor 1	29	0.005	0.679
CRP	C-reactive protein	28	0.008	0.679
ESR1	Estrogen receptor	28	0.008	0.671
MMP2	72 kDa type IV collagenase	28	0.004	0.654
TGFB1	Transforming growth factor beta-1 proprotein	28	0.003	0.671
HMOX1	Heme oxygenase 1	27	0.002	0.663
SOD1	Superoxide dismutase [Cu-Zn]	27	0.013	0.671
IFNG	Interferon gamma	25	0.001	0.631
ICAM1	Intercellular adhesion molecule 1	24	0.000	0.624
PTEN	Phosphatidylinositol 3,4,5-trisphosphate 3-phosphatase and dual-specificity protein phosphatase	24	0.005	0.624
F2	Prothrombin	23	0.010	0.639
IGF2	Insulin-like growth factor II	21	0.005	0.616
ACHE	Acetylcholinesterase	20	0.031	0.609
CD40LG	CD40 ligand	20	0.002	0.596
NOS2	Nitric oxide synthase, inducible	20	0.000	0.596
NFE2L2	Nuclear factor erythroid 2-related factor 2	19	0.001	0.589
GSK3B	Glycogen synthase kinase-3 beta	18	0.001	0.596
MAPT	Microtubule-associated protein tau	16	0.007	0.589
GJA1	Gap junction alpha-1 protein	16	0.000	0.570
BACE1	Beta-secretase 1	15	0.002	0.582
CTSD	Cathepsin D	14	0.003	0.570
BCHE	Cholinesterase	14	0.016	0.564
PON1	Serum paraoxonase/arylesterase 1	13	0.001	0.564
SLC6A4	Sodium-dependent serotonin transporter	11	0.014	0.546
SLC6A3	Sodium-dependent dopamine transporter	11	0.010	0.552
TTR	Transthyretin	10	0.001	0.552
ESR2	Estrogen receptor beta	10	0.000	0.535
ADORA2A	Adenosine receptor A2a	10	0.001	0.546
HTR2A	5-hydroxytryptamine receptor 2A	8	0.004	0.530
DRD4	D (4) dopamine receptors	8	0.003	0.530
BCL2	Apoptosis regulator Bcl-2	7	0.000	0.515
HTR1A	5-hydroxytryptamine receptor 1A	7	0.001	0.473
INSR	Insulin receptor	7	0.000	0.510
MAOA	Amine oxidase [flavin-containing] A	7	0.001	0.417
GBA	Lysosomal acid glucosylceramidase	7	0.000	0.505
CHRNA7	Neuronal acetylcholine receptor subunit alpha-7	7	0.001	0.473
RUNX1T1	Protein CBFA2T1	2	0.000	0.457

**Table 3:** The binding free energy of 20 small molecules with top 5 genes.

Affinity (kcal/mol)	ALB	AKT1	IL6	CASP3	TNF
24-epicampesterol	−9.3	−7.7	−6.5	−7.1	−7.1
Linoleyl acetate	−7.7	−6.2	−4.6	−5.7	−4.4
Poriferast-5-en-3beta-ol	−9.8	−7.7	−7.6	−7.0	−7.0
DFV	−7.8	−7.6	−7.0	−6.9	−6.1
Chryseriol	−9.0	−8.5	−7.1	−7.4	−6.5
8-lsopentenyl-kaempferol	−8.4	−8.5	−6.9	−7.8	−6.8
Sitosterol	−9.7	−8.9	−7.4	−7.4	−7.4
Kaempferol	−8.6	−8.0	−6.8	−7.2	−6.5
Olivil	−8.5	−8.6	−6.3	−7.1	−6.5
Anhydroicaritin	−8.6	−8.5	−7.0	−7.8	−6.6
Yinyanghuo A	−10.0	−9.7	−6.7	−8.4	−8.0
Yinyanghuo C	−11.3	−9.4	−7.7	−9.2	−7.6
Yinyanghuo E	−11.1	−9.6	−7.6	−9.3	−7.3
8-(3-methylbut-2-enyl)-2-phenyl-chromone	−10.5	−9.2	−7.2	−7.6	−7.3
1,2-bis(4-hydroxy-3-methoxyphenyl) propane-1,3-diol	−7.9	−7.5	−7.0	−6.2	−6.2
Icariin	−9.2	−9.3	−6.8	−8.2	−6.6
Icariside A7	−8.3	−9.2	−7.0	−7.6	−7.1
Luteolin	−9.4	−8.5	−7.1	−7.8	−6.7
Magnograndiolide	−7.8	−8.2	−6.4	−6.8	−6.1
Quercetin	−8.8	−8.2	−6.8	−7.3	−6.5

## Data Availability

The data used in the study are available upon request to the corresponding author.

## Supplementary Materials

Supplementary Materials The characteristics of 130 chemical ingredients in Epimedium are shown in Supplementary Table 1. The compound-related targets were selected from TCMSP; SwissTarget and PharmMapper are displayed in Supplementary Table 2. The acknowledged targets of mild cognitive impairment and Alzheimer's disease are listed in Supplementary Table 3. The detailed information about GO/KEGG enrichment analysis is presented in Supplementary Tables 4 and 5.
