# Supplementary material for: A Network Pharmacology Approach to Estimate Potential Targets of the Active Ingredients of Epimedium for Alleviating Mild Cognitive Impairment and Treating Alzheimer's Disease
Source: Evid Based Complement Alternat Med. 2021 Jan 28;2021:2302680. doi: 10.1155/2021/2302680 (PMC7861915; doi:10.1155/2021/2302680)
Supplement: Supplementary Materials — The characteristics of 130 chemical ingredients in Epimedium are shown in Supplementary Table 1. The compound-related targets were selected from TCMSP; SwissTarget and PharmMapper are displayed in Supplementary Table 2. The acknowledged targets of mild cognitive impairment and Alzheimer's disease are listed in Supplementary Table 3. The detailed information about GO/KEGG enrichment analysis is presented in Supplementary Tables 4 and 5. [file 2302680.f1.docx]

TABLE 1: The characteristics of chemical ingredients in Epimedium.

| Mol ID | Molecule Name | Molecular Weight | OB(％) | Caco-2 | BBB | DL | HL |
| --- | --- | --- | --- | --- | --- | --- | --- |
| MOL000118 | (L)-alpha-Terpineol | 154.28 | 48.8 | 1.39 | 1.72 | 0.03 | 11.35 |
| MOL000120 | dec-2-enal | 154.28 | 18.55 | 1.4 | 1.59 | 0.02 | / |
| MOL000130 | CAM | 152.26 | 67.17 | 1.29 | 1.71 | 0.05 | 11.34 |
| MOL001510 | 24-epicampesterol | 400.76 | 37.58 | 1.43 | 1.15 | 0.71 | 4.5 |
| MOL001579 | germacrene | 208.43 | 15.06 | 1.82 | 2.06 | 0.06 | / |
| MOL001600 | copaene | 204.39 | 29.47 | 1.81 | 2.04 | 0.12 | / |
| MOL001640 | NON | 172.3 | 26.74 | 0.96 | 1.06 | 0.03 | / |
| MOL001645 | Linoleyl acetate | 308.56 | 42.1 | 1.36 | 1.08 | 0.2 | 7.48 |
| MOL001707 | 24190-29-2 | 192.33 | 21.67 | 1.38 | 1.54 | 0.05 | / |
| MOL001771 | poriferast-5-en-3beta-ol | 414.79 | 36.91 | 1.45 | 1.14 | 0.75 | 5.07 |
| MOL001789 | isoliquiritigenin | 256.27 | 85.32 | 0.44 | -0.41 | 0.15 | 17.66 |
| MOL001792 | DFV | 256.27 | 32.76 | 0.51 | -0.29 | 0.18 | 17.89 |
| MOL001972 | Pulegone | 152.26 | 51.6 | 1.39 | 1.74 | 0.03 | 11.19 |
| MOL000198 | (R)-linalool | 154.28 | 39.8 | 1.33 | 1.36 | 0.02 | 6.48 |
| MOL000203 | Izosafrol | 162.2 | 56.92 | 1.45 | 1.31 | 0.05 | 5.14 |
| MOL002040 | (1S ,4R)-fenchone | 152.26 | 72.64 | 1.35 | 1.74 | 0.05 | 11.25 |
| MOL000205 | (6R)-6-isopropyl-3-methyl-1-cyclohex-2-enone | 152.26 | 53.88 | 1.3 | 1.55 | 0.03 | 11.29 |
| MOL000207 | Methyleugenol | 178.25 | 73.36 | 1.47 | 1.41 | 0.04 | 2.92 |
| MOL002083 | tricin | 330.31 | 27.86 | 0.51 | -0.6 | 0.34 | / |
| MOL002085 | alpha-Cubebene | 204.39 | 16.73 | 1.83 | 2.1 | 0.11 | / |
| MOL002307 | 20-Hexadecanoylingenol | 586.94 | 28.2 | 0.3 | -0.04 | 0.68 | / |
| MOL002361 | Terragon | 148.22 | 36.59 | 1.72 | 1.83 | 0.03 | 1.79 |
| MOL000244 | ()-Borneol | 154.28 | 81.8 | 1.22 | 1.47 | 0.05 | 11.36 |
| MOL002509 | Ginkgetin | 566.54 | 22.19 | 0.12 | -1.48 | 0.59 | / |
| MOL002511 | Isoginkgetin | 566.54 | 21.56 | 0.02 | -1.69 | 0.58 | / |
| MOL000263 | oleanolic acid | 456.78 | 29.02 | 0.59 | 0.07 | 0.76 | / |
| MOL002689 | 3,4,5-Trimethoxytoluene | 182.24 | 23.73 | 1.32 | 1.25 | 0.04 | / |
| MOL002697 | junipene | 204.39 | 44.07 | 1.82 | 2.14 | 0.11 | 12.2 |
| MOL002891 | magnoflorine | 342.45 | 0.48 | 1.07 | 0.69 | 0.55 | / |
| MOL002929 | salidroside | 300.34 | 7.01 | -0.82 | -1.41 | 0.2 | / |
| MOL002930 | Tyrosol | 138.18 | 33.81 | 0.65 | 0.25 | 0.02 | -2.55 |
| MOL003030 | Ginnol | 424.89 | 11.33 | 1.46 | 0.81 | 0.43 | / |
| MOL003044 | Chryseriol | 300.28 | 35.85 | 0.39 | -0.53 | 0.27 | 16.31 |
| MOL000305 | lauric acid | 200.36 | 23.59 | 1.02 | 1.1 | 0.04 | / |
| MOL003097 | Flavone der. | 298.31 | 27.12 | 0.83 | 0.03 | 0.27 | / |
| MOL000325 | (2R,3R)-2-(3,4-dimethoxyphenyl)-7-methoxy-3-methyl-5-[(E)-prop-1-enyl]-2,3-dihydrobenzofuran | 340.45 | 24.19 | 1.48 | 0.93 | 0.35 | / |
| MOL003518 | Vetol | 126.12 | 53.23 | 0.64 | 0.7 | 0.02 | 11.82 |
| MOL003520 | Damascenone | 190.31 | 36.43 | 1.34 | 1.6 | 0.05 | 8.2 |
| MOL003542 | 8-Isopentenyl-kaempferol | 354.38 | 38.04 | 0.53 | -0.49 | 0.39 | 15.37 |
| MOL003547 | Azaron | 208.28 | 38.39 | 1.42 | 1.18 | 0.06 | 4.55 |
| MOL000357 | Sitogluside | 576.95 | 20.63 | -0.14 | -0.93 | 0.62 | / |
| MOL000359 | sitosterol | 414.79 | 36.91 | 1.32 | 0.87 | 0.75 | 5.37 |
| MOL000399 | Docosanoate | 340.66 | 15.69 | 1.21 | 0.91 | 0.26 | / |
| MOL000422 | kaempferol | 286.25 | 41.88 | 0.26 | -0.55 | 0.24 | 14.74 |
| MOL004363 | (Z)-heptadec-3-ene | 238.51 | 20.24 | 1.84 | 1.99 | 0.08 | / |
| MOL004364 | 3,5-Dimethoxytoluene | 152.21 | 20.87 | 1.52 | 1.49 | 0.03 | / |
| MOL004365 | Isomenthol | 156.3 | 55.3 | 1.35 | 1.63 | 0.03 | 10.88 |
| MOL004366 | Octyl formate | 158.27 | 53.32 | 1.19 | 1.24 | 0.02 | 6.99 |
| MOL004367 | olivil | 376.44 | 62.23 | -0.16 | -0.75 | 0.41 | 2.27 |
| MOL004368 | Hyperin | 464.41 | 6.94 | -1.42 | -2.08 | 0.77 | / |
| MOL004369 | quercetin-3-rhamnooside | 448.41 | 2.61 | -1.12 | -1.83 | 0.74 | / |
| MOL000437 | Hirsutrin | 464.41 | 1.86 | -1.66 | -2.31 | 0.77 | / |
| MOL004370 | Robinetin | 302.25 | 6.35 | -0.13 | -0.79 | 0.28 | / |
| MOL004371 | rouhuoside | 824.86 | 3.62 | -3.58 | -4.81 | 0.31 | / |
| MOL004372 | Sagittatoside A | 676.73 | 8.5 | -1.56 | -2.32 | 0.57 | / |
| MOL004373 | Anhydroicaritin | 368.41 | 45.41 | 0.72 | 0.01 | 0.44 | 15.01 |
| MOL004374 | sagittatoside B | 646.7 | 5.58 | -1.18 | -2.16 | 0.64 | / |
| MOL004375 | Trifolin | 448.41 | 3.1 | -1.17 | -1.85 | 0.74 | / |
| MOL004376 | wanepimedoside A | 678.75 | 6.54 | -1.57 | -2.45 | 0.58 | / |
| MOL004377 | wanepimedoside_qt | 386.43 | 6.03 | 0.22 | -0.62 | 0.48 | / |
| MOL004378 | Wushanicariin | 530.57 | 5.49 | -1.11 | -2.17 | 0.86 | / |
| MOL004379 | wushanicariin_qt | 368.41 | 23.36 | 0.56 | -0.36 | 0.46 | / |
| MOL004380 | C-Homoerythrinan,1,6-didehydro-3,15,16-trimethoxy-(3.beta.)- | 329.48 | 39.14 | 1.02 | 0.68 | 0.49 | 6.58 |
| MOL004381 | Besigomsin | 416.51 | 28.52 | 0.63 | 0.25 | 0.78 | / |
| MOL004382 | Yinyanghuo A | 420.49 | 56.96 | 0.38 | -0.49 | 0.77 | 14.44 |
| MOL004383 | Yinyanghuo B | 422.51 | 1.07 | 0.24 | -1.14 | 0.6 | / |
| MOL004384 | Yinyanghuo C | 336.36 | 45.67 | 0.75 | -0.11 | 0.5 | 15.74 |
| MOL004385 | Yinyanghuo D | 338.38 | 13.99 | 0.61 | -0.53 | 0.38 | / |
| MOL004386 | Yinyanghuo E | 352.36 | 51.63 | 0.51 | -0.54 | 0.55 | 15.47 |
| MOL004387 | Yixinoside A | 1107.49 | 6.13 | -4.09 | -5.42 | 0.04 | / |
| MOL004388 | 6-hydroxy-11,12-dimethoxy-2,2-dimethyl-1,8-dioxo-2,3,4,8-tetrahydro-1H-isochromeno[3,4-h]isoquinolin-2-ium | 370.41 | 60.64 | 0.34 | -0.12 | 0.66 | 1.22 |
| MOL004389 | 3-Hexenyl-beta-glucopyranoside | 262.34 | 12.12 | -0.57 | -0.79 | 0.11 | / |
| MOL004390 | 5,7,4'-trihydroxy8,3'-diprenylflavone | 406.51 | 1.02 | 0.93 | -0.16 | 0.59 | / |
| MOL004391 | 8-(3-methylbut-2-enyl)-2-phenyl-chromone | 290.38 | 48.54 | 1.53 | 0.99 | 0.25 | 18.73 |
| MOL004392 | acuminatoside | 985.05 | 3.01 | -4.04 | -5.25 | 0.15 | / |
| MOL004393 | anhydroicaritin | 368.41 | 28.27 | 0.77 | 0.03 | 0.59 | / |
| MOL004394 | Anhydroicaritin-3-O-alpha-L-rhamnoside | 676.73 | 41.58 | -1.59 | -2.89 | 0.61 | 16.23 |
| MOL004395 | artonin U | 352.41 | 19.33 | 0.86 | 0.22 | 0.39 | / |
| MOL004396 | 1,2-bis(4-hydroxy-3-methoxyphenyl)propan-1,3-diol | 320.37 | 52.31 | 0 | -0.87 | 0.22 | 2.18 |
| MOL004397 | baohuoside Ⅵ | 822.89 | 6.06 | -3.06 | -3.87 | 0.32 | / |
| MOL004398 | 3,5,7-Trihydroxy-4'-methoxyl-8-prenylflavone-3-O-rhamnopyranoside | 514.57 | 3.7 | -0.55 | -1.24 | 0.84 | / |
| MOL004399 | Baohuoside VI | 822.89 | 4.94 | -2.92 | -3.89 | 0.31 | / |
| MOL004400 | Bilobanol | 234.37 | 14.22 | 1.16 | 0.96 | 0.09 | / |
| MOL004401 | bilobetin | 552.51 | 7.27 | -0.14 | -1.83 | 0.63 | / |
| MOL004402 | brevicornin | 400.46 | 14.09 | 0.42 | -0.1 | 0.52 | / |
| MOL004403 | caohuoside B | 965.01 | 3.02 | -2.94 | -3.73 | 0.19 | / |
| MOL004404 | caohuoside D | 562.62 | 24.89 | -1.02 | -2.07 | 0.83 | / |
| MOL004405 | 3-[(2S,3R,4R,5R,6S)-4,5-dihydroxy-6-methyl-3-[(2S,3R,4S,5S,6R)-3,4,5-trihydroxy-6-methyl-tetrahydropyran-2-yl]oxy-tetrahydropyran-2-yl]oxy-5-hydroxy-2-(4-methoxyphenyl)-8-(3-methylbut-2-enyl)-7-[(2S,3R,4S,5S,6R)-3,4,5-trihydroxy-6-methylol-tetrahydropyran | 822.89 | 6.06 | -2.64 | -3.91 | 0.32 | / |
| MOL004406 | 2,15-Hexadecanedione | 254.46 | 8.15 | 0.88 | 0.38 | 0.1 | / |
| MOL004407 | Epimedin B | 792.86 | 8.65 | -2.89 | -3.91 | 0.34 | / |
| MOL004408 | Epimedin C_qt | 352.41 | 5.87 | 0.82 | -0.06 | 0.4 | / |
| MOL004409 | Epimedin C | 790.89 | 16.29 | -2.5 | -3.48 | 0.34 | / |
| MOL004410 | epimedokoreanone A | 142.12 | 59.85 | 0.3 | 0.11 | 0.03 | 11.79 |
| MOL004411 | (2S,3S)-3,5-dihydroxy-2-(4-hydroxyphenyl)-8-(3-methylbut-2-enyl)-7-[(2S,3R,4S,5S,6R)-3,4,5-trihydroxy-6-methylol-tetrahydropyran-2-yl]oxy-chroman-4-one | 518.56 | 3.58 | -1.39 | -2.36 | 0.84 | / |
| MOL004413 | epimedoside C | 516.54 | 2.67 | -0.96 | -2.04 | 0.84 | / |
| MOL004414 | epimedoside D | 794.83 | 5.49 | -2.71 | -3.82 | 0.34 | / |
| MOL004415 | Epimedoside E | 794.83 | 5.49 | -3.15 | -4.05 | 0.35 | / |
| MOL004417 | epimedoside | 760.81 | 14.32 | -1.4 | -2.34 | 0.41 | / |
| MOL004418 | DOB | 154.13 | 88.18 | 0.26 | 0.01 | 0.04 | 11.88 |
| MOL004419 | globulol | 222.41 | 19.94 | 1.31 | 1.43 | 0.12 | / |
| MOL004420 | Hentriacontanol-6 | 452.95 | 11.06 | 1.33 | 0.68 | 0.54 | / |
| MOL004421 | hexandraside D | 822.89 | 5.44 | -2.56 | -3.52 | 0.32 | / |
| MOL004422 | Hexandraside E | 678.7 | 13.56 | -2.3 | -3.47 | 0.6 | / |
| MOL004423 | hexandraside F | 838.89 | 3.67 | -3.37 | -4.36 | 0.3 | / |
| MOL004424 | Icaride A2 | 436.5 | 4.76 | -0.19 | -1.28 | 0.54 | / |
| MOL004425 | Icariin | 676.73 | 41.58 | -1.82 | -3 | 0.61 | 19.93 |
| MOL004426 | Icariresinol | 433.47 | 7.13 | -0.53 | -1.17 | 0.53 | / |
| MOL004427 | Icariside A7 | 462.49 | 31.91 | -0.65 | -1.56 | 0.86 | 2.84 |
| MOL004428 | 3,4,6-trimethoxyphenanthrene-2,7-diol | 300.33 | 24.46 | 0.92 | 0.21 | 0.3 | / |
| MOL004429 | icariside C1 | 418.59 | 5.12 | -1.11 | -1.87 | 0.37 | / |
| MOL004430 | icariside I | 530.57 | 21.88 | -0.93 | -1.79 | 0.85 | / |
| MOL004431 | icariside II | 514.57 | 3.7 | -0.21 | -0.91 | 0.84 | / |
| MOL004432 | 4H-1-Benzopyran-4-one, 3-((6-deoxy-alpha-L-mennopyranosyl)oxy)-5,7-dihydroxy-2-(4-hydroxyphenyl)-8-(3-methyl-2-butenyl)- | 500.54 | 4.75 | -0.7 | -1.39 | 0.82 | / |
| MOL004433 | 3-[(2S,3R,4S,5S,6R)-4,5-dihydroxy-6-methylol-3-[(2S,3R,4R,5R,6S)-3,4,5-trihydroxy-6-methyl-tetrahydropyran-2-yl]oxy-tetrahydropyran-2-yl]oxy-5,7-dihydroxy-2-(4-hydroxyphenyl)-8-(3-methylbut-2-enyl)chromone | 662.7 | 2.93 | -2.08 | -3.02 | 0.61 | / |
| MOL004434 | Ikarisoside C | 822.89 | 4.96 | -3.58 | -4.36 | 0.31 | / |
| MOL004435 | Ikarisoside F | 632.67 | 1.95 | -1.83 | -2.7 | 0.67 | / |
| MOL004436 | Ikshusterol | 430.79 | 9 | 0.88 | 0.26 | 0.79 | / |
| MOL004437 | Lespedin | 578.57 | 7.97 | -1.98 | -3.08 | 0.79 | / |
| MOL004438 | korepimedoside A | 748.8 | 14.93 | -1.74 | -2.3 | 0.44 | / |
| MOL004439 | korepimedoside B | 965.01 | 3.02 | -2.76 | -3.9 | 0.19 | / |
| MOL000472 | emodin | 270.25 | 24.4 | 0.22 | -0.66 | 0.24 | / |
| MOL000478 | Eucarvone | 150.24 | 53.14 | 1.35 | 1.65 | 0.03 | 11.45 |
| MOL000561 | Astragalin | 448.41 | 14.03 | -1.34 | -1.97 | 0.74 | / |
| MOL000006 | luteolin | 286.25 | 36.16 | 0.19 | -0.84 | 0.25 | 15.94 |
| MOL000622 | Magnograndiolide | 266.37 | 63.71 | 0.02 | -0.24 | 0.19 | 3.17 |
| MOL000667 | 1-hexanol | 102.2 | 22.04 | 1.08 | 1.21 | 0.01 | / |
| MOL000695 | patchouli alcohol | 222.41 | 101.96 | 1.4 | 1.58 | 0.14 | 12.64 |
| MOL000740 | (+)-Cycloolivil | 376.44 | 24.5 | -0.29 | -1.07 | 0.42 | / |
| MOL000008 | apigenin | 270.25 | 23.06 | 0.43 | -0.61 | 0.21 | / |
| MOL000098 | quercetin | 302.25 | 46.43 | 0.05 | -0.77 | 0.28 | 14.4 |

TABLE 2: The compound-related target in Epimedium.

| Database (Number of target) | Target name (Gene name) |
| --- | --- |
| TCMSP (204) | ACACA ACHE ACP3 ADCY2 ADRA1A ADRA1B ADRA1D ADRA2A ADRA2B ADRB1 ADRB2 AHR AHSA1 AKR1C3 AKT1 ALOX5 APP AR BAX BCL2 BCL2L1 BIRC5 CALM1 CALM2 CALM3 CASP3 CASP7 CASP8 CASP9 CAV1 CCL2 CCNA2 CCNB1 CCND1 CD40LG CDKN1A CHEK1 CHEK2 CHRM1 CHRM2 CHRM3 CHRM4 CHRM5 CHRNA2 CHRNA7 CHUK CLDN4 COL1A1 CRP CTSD CXCL10 CXCL11 CXCL2 CXCL8 CYP1A1 CYP1A2 CYP1B1 CYP3A4 DCAF5 DIO1 DPP4 DRD1 DRD5 DUOX2 E2F1 E2F2 EGF EGFR EIF6 ELK1 ERBB2 ERBB3 ESR1 ESR2 F3 F7 FOS GABRA1 GABRA2 GABRA3 GABRA6 GABRE GABRG3 GJA1 GRIA2 GSK3B GSTM1 GSTM2 GSTP1 HAS2 HIF1A HK2 HMOX1 HRH1 HSF1 HSP90AB1 HSPB1 HTR1A HTR1B HTR2A HTR2C HTR3A ICAM1 IFNG IGF2 IGFBP3 IKBKB IL10 IL1A IL1B IL2 IL4 IL6 INSR IRF1 JUN KCNH2 KCNMA1 KDR MAOB MAPK1 MAPK14 MAPK8 MCL1 MDM2 MET MGAM MMP1 MMP2 MMP3 MMP9 MPO MYC NCF1 NCOA1 NCOA2 NFE2L2 NFKBIA NKX3-1 NOS2 NOS3 NPEPPS NR1I2 NR1I3 NR3C2 NUF2 ODC1 OPRD1 OPRM1 PARP1 PCNA PCOLCE PDE3A PGR PIK3CG PIM1 PKIA PLAT PLAU PON1 POR PPARA PPARD PPARG PPP3CA PRKCA PRKCB PRSS1 PSMD3 PTEN PTGER3 PTGES PTGS1 PTGS2 PYGM RAF1 RASA1 RASSF1 RB1 RELA RUNX1T1 RUNX2 RXRA RXRB SCN5A SELE SERPINE1 SLC2A4 SLC6A2 SLC6A3 SLC6A4 SLPI SOD1 SPP1 STAT1 TGFB1 THBD TNF TOP1 TP53 TYR VCAM1 VEGFA XDH |
| SwissTarget (108) | ABCB1 ABCC1 ABCG2 ACHE ADORA1 ADORA2A AHR AKR1A1 AKR1B1 AKR1B10 AKR1C1 AKR1C2 AKR1C3 AKR1C4 AKT1 ALK ALOX12 ALOX15 ALOX5 APEX1 APP ARG1 AURKB AVPR2 AXL BACE1 CA1 CA12 CA13 CA14 CA2 CA3 CA4 CA5A CA6 CA7 CA9 CAMK2B CCNB1 CCNB2 CCNB3 CD38 CDK1 CDK2 CDK5 CDK5R1CDK6 CSNK2A1 CXCR1 CYP17A1 CYP19A1 CYP1B1 DAPK1 DRD4 EGFR ESR1 ESR2 ESRRA F2 FLT3 GLO1 GPR35 GSK3B HMGCR HSD17B1 HSD17B2 IGF1R INSR KDM4E KDR MAOA MAPT MET MMP12 MMP13 MMP2 MMP3 MMP9 MPG MPO MYLK NEK2 NEK6NOX4 NPC1L1 NR1H3 NUAK1 PARP1 PDE5A PIK3CG PIK3R1 PIM1 PKN1 PLA2G1B PLK1 PTK2 PTPRS PYGL RORC SLC22A12 SRC SYK TNKS TNKS2 TOP1 TTR TYR XDH |
| PharmMapper (84) | ADAM17 AKR1A1 AKR1B1 AKR1B10 AKR1C2 AKR1C3 ALB AMY1A AMY1B AMY1C ANXA5 APOA2 AR BACE1 BCHE BMP2 BMP7 CA1 CA12 CA2 CASP3 CASP7 CCNA2 CDK5R1 CES1 CFB CHEK1 CMA1 CTSD CTSV CYP19A1 DDX6 EGFR EPHB4 ESR1 ESR2 ESRRG F2 FCAR FKBP1A GBA GC GSR GSTP1 HCK HSD17B11 HSP90AA1 HSPA8 ICAM2 ITGAL KDR KIF11 LTA4H MAOB MAPK1 MAPK10 MAPK14 MAPK8 MAPKAPK2 METAP1 MMP13 MMP3 MTAP NQO2 NUDT9 PAH PGR PIM1 PLG PNMT PNP PPARG PPIA PYGL QPCT RORA SEC14L2 SELP SHBG STS TGFBR2 THRB TREM1 TTR |
| Final Identified Target  in Epimedium (337) | ABCB1 ABCC1 ABCG2 ACACA ACHE ACP3 ADAM17 ADCY2 ADORA1 ADORA2A ADRA1A  ADRA1B ADRA1D ADRA2A ADRA2B ADRB1 ADRB2 AHR AHSA1 AKR1A1 AKR1B1  AKR1B10 AKR1C1 AKR1C2 AKR1C3 AKR1C4 AKT1 ALB ALK ALOX12 ALOX15 ALOX5 AMY1A  AMY1B AMY1C ANXA5 APEX1 APOA2 APP AR ARG1 AURKB AVPR2 AXL BACE1 BAX  BCHE BCL2 BCL2L1 BIRC5 BMP2 BMP7 CA1 CA12 CA13 CA14 CA2 CA3 CA4 CA5A CA6  CA7 CA9 CALM1 CALM2 CALM3 CAMK2B CASP3 CASP7 CASP8 CASP9 CAV1 CCL2  CCNA2 CCNB1 CCNB2 CCNB3 CCND1 CD38 CD40LG CDK1 CDK2 CDK5 CDK5R1 CDK6  CDKN1A CES1 CFB CHEK1 CHEK2 CHRM1 CHRM2 CHRM3 CHRM4 CHRM5 CHRNA2 CHRNA7  CHUK CLDN4 CMA1 COL1A1 CRP CSNK2A1 CTSD CTSV CXCL10 CXCL11 CXCL2 CXCL8  CXCR1 CYP17A1 CYP19A1 CYP1A1 CYP1A2 CYP1B1 CYP3A4 DAPK1 DCAF5 DDX6 DIO1  DPP4 DRD1 DRD4 DRD5 DUOX2 E2F1 E2F2 EGF EGFR EIF6 ELK1 EPHB4 ERBB2  ERBB3 ESR1 ESR2 ESRRA ESRRG F2 F3 F7 FCAR FKBP1A FLT3 FOS GABRA1 GABRA2  GABRA3 GABRA6 GABRE GABRG3 GBA GC GJA1 GLO1 GPR35 GRIA2 GSK3B GSR  GSTM1 GSTM2 GSTP1 HAS2 HCK HIF1A HK2 HMGCR HMOX1 HRH1 HSD17B1 HSD17B11  HSD17B2 HSF1 HSP90AA1 HSP90AB1 HSPA8 HSPB1 HTR1A HTR1B HTR2A HTR2C HTR3A  ICAM1 ICAM2 IFNG IGF1R IGF2 IGFBP3 IKBKB IL10 IL1A IL1B IL2 IL4 IL6 INSR IRF1  ITGAL JUN KCNH2 KCNMA1 KDM4E KDR KIF11 LTA4H MAOA MAOB MAPK1 MAPK10  MAPK14 MAPK8 MAPKAPK2 MAPT MCL1 MDM2 MET METAP1 MGAM MMP1 MMP12 MMP13  MMP2 MMP3 MMP9 MPG MPO MTAP MYC MYLK NCF1 NCOA1 NCOA2 NEK2  NEK6 NFE2L2 NFKBIA NKX3-1 NOS2 NOS3 NOX4 NPC1L1 NPEPPS NQO2 NR1H3 NR1I2  NR1I3 NR3C2 NUAK1 NUDT9 NUF2 ODC1 OPRD1 OPRM1 PAH PARP1 PCNA PCOLCE PDE3A  PDE5A PGR PIK3CG PIK3R1 PIM1 PKIA PKN1 PLA2G1B PLAT PLAU PLG PLK1 PNMT  PNP PON1 POR PPARA PPARD PPARG PPIA PPP3CA PRKCA PRKCB PRSS1 PSMD3 PTEN  PTGER3 PTGES PTGS1 PTGS2 PTK2 PTPRS PYGL PYGM QPCT RAF1 RASA1 RASSF1 RB1  RELA RORA RORC RUNX1T1 RUNX2 RXRA RXRB SCN5A SEC14L2 SELE SELP  SERPINE1 SHBG SLC22A12 SLC2A4 SLC6A2 SLC6A3 SLC6A4 SLPI SOD1 SPP1 SRC STAT1  STS SYK TGFB1 TGFBR2 THBD THRB TNF TNKS TNKS2 TOP1 TP53 TREM1 TTR TYR  VCAM1 VEGFA XDH |

TABLE 3: The acknowledged target of AD and MCI.

| Database  Disease (Number of target) | Target name (Gene name) |
| --- | --- |
| GeneCards  AD (546) | A2M ABCA1 ABCA4 ABCA7 ABCB1 ABL1 ACE ACHE ACTC1 AD10 AD11 AD12 AD13 AD14 AD15 AD16 AD17 AD5 AD6 AD7 AD8 ADAM10 ADAM17 ADAM19 ADIPOQ ADNP ADORA2A AFP AGER AGRN AGTR1 AKT1 ALB ALDH2 ALOX5 AOC3 APAF1 APBA1 APBA2 APBA3 APBB1 APBB2 APBB3 APC APEX1 APH1A APLP1 APLP2 APOA1 APOB APOC1 APOD APOE APP AQP4 ARSA ATN1 ATP13A2 ATP2A2 ATP5F1A ATP7B ATXN1 ATXN2 ATXN3 BACE1 BACE2 BAD BAX BCHE BDNF BIN1 BLMH BLVRB BPTF C1R C4A C9orf72 CACNA1A CACNA1C CALB1 CALB2 CALHM1 CALM1 CALM2 CALM3 CAPN1 CAPN2 CASP1 CASP2 CASP3 CASP6 CASP7 CASP8 CASP9 CAST CAT CAV1 CAV3 CBS CCK CCL2 CCL3 CCL5 CCR5 CD28 CD33 CD40 CD40LG CDK1 CDK4 CDK5 CDK5R1 CDK5RAP2 CDKN2A CDKN2B-AS1 CDKN3 CETP CFH CH25H CHAT CHCHD10 CHI3L1 CHMP2B CHRM1 CHRNA4 CHRNA7 CHRNB2 CIB1 CLN5 CLSTN1 CLU CNR1 CNTF COL25A1 COL4A1 COMT COX5A COX6B1 CP CR1 CREB1 CRH CRP CRYAB CSF1R CSNK1D CST3 CSTB CTNNA3 CTNNB1 CTSB CTSD CX3CR1 CXCL12 CXCL8 CXCR4 CYBB CYCS CYP2D6 CYP46A1 DBH DBN1 DCHS2 DCTN1 DHCR24 DKK1 DKK3 DLG4 DLST DNM1L DNMBP DNMT1 DOCK3 DPYSL2 DRD1 DRD2 DRD3 DRD4 DYNC1H1 DYRK1A ECE1 EDN1 EDNRB EGF EGFR EGR1 EIF2AK2 EIF2AK3 EIF2S1 ELANE EPM2A ERBB2 ERBB4 ESR1 ESR2 EXOC3L2 F2 FABP3 FAS FGFR1 FLG FMR1 FRMD4A FUS FYN GAA GAD1 GAL GAP43 GAPDH GATA1 GBA GCG GDNF GFAP GHRL GJA1 GJB1 GLRX GLUL GNAQ GPC1 GRB2 GRIA1 GRIA2 GRIA3 GRIN1 GRIN2A GRIN2B GRIN2C GRK2 GRN GSAP GSK3A GSK3B GSN GSR GSS GSTO1 GSTP1 HBA1 HCRT HFE HLA-B HLA-DQB1 HLA-DRB1 HLA-G HMBS HMGB1 HMGCR HMOX1 HNRNPA1 HNRNPA2B1 HRH2 HSD17B10 HSPA1A HSPA5 HSPA8 HSPG2 HTR1A HTR2A HTR2C HTR6 HTRA1 HTT ICAM1 IDE IFNG IGF1 IGF2R IL10 IL1A IL1B IL1RN IL2 IL2RA IL4 IL6 INS INSR ITM2B ITPR1 ITPR3 JAG1 JAK2 JAK3 JCAD JPH3 JUN KIDINS220 KLK6 KRAS KRT14 LDHA LDLR LEP LMNA LOX LPA LPL LRAT LRP1 LRP2 LRP8 LRPAP1 LRRK2 LTA MAOA MAOB MAP1B MAP2 MAP2K1 MAP3K5 MAPK1 MAPK10 MAPK14 MAPK3 MAPK8 MAPK8IP1 MAPT MARK1 MARK4 MECP2 MFN2 MIAT MIR106B MIR107 MIR125A MIR128-1 MIR132 MIR146A MIR155 MIR15A MIR181C MIR197 MIR21 MIR210 MIR22 MIR298 MIR29A MIR29B1 MIR320A MIR328 MIR34A MIR93 MIRLET7I MME MMP2 MMP3 MMP9 MPO MPZ MS4A6A MSR1 MT3 MT-ATP6 MT-CO1 MT-CO2 MTHFR MT-ND1 MT-ND2 MTOR MTR  MT- RNR2 MTRR MYC MYOCD NAE1 NCSTN NDUFS3 NDUFS4 NEFH NEFL NEFM NGF NGFR NHLRC1 NOD2 NOS1 NOS2 NOS3 NOTCH1 NOTCH3 NPC1 NPC2 NPPB NPY NQO1 NR3C1 NRG1 NRGN NTF3 NTF4 NTRK1 NTRK2 NTRK3 NTS OGDH OGG1 OGT OLR1 OXT P2RX7 PARK7 PCDH11X PCSK1N PDGFB PDSS1 PDYN PFKM PICALM PIN1 PINK1 PKD1 PLA2G2A PLA2G4A PLA2G6 PLAT PLAU PLCB1 PLCB4 PLD3 PLP1 PLTP POLG POMC PON1 PON2 PPARA PPARG PPP3CA PRKCA PRKCB PRKCZ PRKN PRND PRNP PRODH PSEN1 PSEN2 PSENEN PSMB8 PTEN PTGS1 PTGS2 PTPA PVALB PZP RAB5A RAB7A RBFOX3 RCAN1 REG1A RELN REST RET RHOA RPS27A RPS6KB1 RTN4 RUNX1 RYR1 S100B SAA1 SEMA3A SERPINA3 SERPINE1 SERPINI1 SIGMAR1 SIRT1 SLC17A5 SLC18A2 SLC18A3 SLC1A2 SLC1A3 SLC2A1 SLC2A3 SLC30A6 SLC5A7 SLC6A3 SLC6A4 SMPD1 SNAP25 SNCA SNCAIP SNCB SNCG SOAT1 SOD1 SOD2 SORL1 SOX10 SPAST SPG21 SPHK2 SPTLC1 SPTLC2 SQSTM1 SRC SST STAT3 STH STMN2 SYNJ1 SYP SYT1 TARDBP TBK1 TBP TF TFAM TFCP2 TGFB1 TGM2 TH THBS4 TLR2 TLR4 TMEM106B TNF TNFRSF11B TNFRSF1A TNFRSF1B TOMM40 TP53 TREM2 TSPO TTBK1 TTR TXN TYROBP UBB UBQLN1 UBQLN2 UCHL1 UNC5C VCP VDAC1 VDR VEGFA VLDLR VPS26A VPS35 VSNL1 VWF WWOX |
| MCI (441) | APOE APP BDNF MAPT COMT TNF SNCA MECP2 TP53 FMR1 IL6 IGF1 INS ALB IL10 SOD1 CACNA1A POLG SLC6A3 C9orf72 MTHFR SPG11 PSEN1 PRNP DRD2 AKT1 TH GJB2 SPG7 GFAP ZFYVE26 NGF FA2H SCN1A CHAT PRODH PLP1 SCN8A SLC6A4 HTT PRKN SLC2A1 PLA2G6 DMD MTOR SPG21 ATP13A2 SPAST TARDBP SYP ERCC6 TLR4 GRIN2B FGFR3 VCP ATXN3 HLA-DRB1 COL2A1 MFN2 MMP9 TTR GBA FBN1 BSCL2 FGFR2 LRRK2 GRIN2A HTR2A NAGLU MAOA FGFR1 NEFL NRXN1 FGF14 ITPR1 SQSTM1 CAT AFG3L2 SPTBN2 FXN LEP CNTNAP2 ARSA DRD3 PSEN2 ATXN2 PNPLA6 F2 CD40LG ATXN1 WASHC5 CTSD COL1A1 CHRNA7 GBA2 FAS GRN KIF5A APTX VWF GJC2 POMC SLC1A2 ARX MAG ATL1 PTEN CP NTRK2 POMGNT1 CCL2 PPARG GDNF NF1 MT-ATP6 GJB1 UCHL1 NPC1 MT-CO1 NTRK1 IGF2 HTR1A PAH ELN TGFB1 STXBP1 DGUOK MT-ND1 GLB1 C19orf12 FUS SHANK3 FKRP NDUFS4 ATRX PTPN11 POMT1 NAGA HLA-DQB1 AP5Z1 KIF1A IDUA C12orf65 ESR1 ATP1A3 BCHE MIR132 SHH DCAF17 EP300 EGF PINK1 LRP5 PSAP SETX LMNA NOTCH3 CST3 SCN2A POMT2 SORL1 PARK7 PCDH19 TNFRSF1A PEX6 TTPA KCNC3 NOS3 FKTN FLNA DBH UBE3A PAX6 AR MT-TL1 USH2A GABRG2 FTL BRCA1 ATM CACNA1C TBX1 GJB6 MT-TK TSC2 HFE SPART TBP GABRA1 ACE DNM1L NLGN4X CRYAA OPA1 REEP1 GLA COASY GABRB3 PANK2 GLI3 ADNP HSPD1 TMEM106B TLR2 GCH1 CRYAB POLR3A MPO GRIA3 DARS2 RELN POGZ PIK3CA GMPPB WFS1 DAG1 CYP2D6 GNAS GJA1 STAT1 ARID1B DRD4 CFH TREX1 BRAF MT- ND6 COL1A2 MED12 ABCC8 TREM2 ATXN7 GRM1 SLC1A3 AIFM1 ERCC4 ACHE SOX10 CHD7 ERCC2 FGF8 ALDH18A1 DLG3 TBC1D24 DISC1 TWNK NIPA1 CRP WDR45 OPA3 SNAP25 SGSH SDHA TTN ATP1A2 MAPK1 GRIN1 PON1 PEX16 MT-CYB TMEM240 ABCA1 PEX10 HMOX1 CLN3 RYR1 SACS APOA1 SEMA3A SPP1 DNMT1 TOMM40 G6PD CSF1R CHMP2B EPRS1 PRRT2 MTR CYCS ANK3 PDYN NOTCH1 KCNJ11 CTNNB1 VPS13A HEPACAM LAMA2 NOS2 APOB CEP290 HLA-B SMARCA2 GHRL PRPS1 PEX1 PCNT IQSEC2 TNFRSF1B C4A AFF2 SLC18A2 RAB39B ACTB IL1B TCF4 CPLX1 CDKL5 POLR3B HPRT1 TBK1 TLR3 NSD1 CRH MT-CO2 KIF1C SYNJ1 TCF20 SDHB SCN9A OTC SLC33A1 PIK3R1 MT-ND4 HRAS SLC25A13 RTN2 SDHD ICAM1 STAT3 GABRB2 GALNS ATR GRIK2 CD40 MPZ CHRNA4 ATXN8OS DMPK BRCA2 RAD51 TFRC JPH3 SUOX PAFAH1B1 BMP4 ERCC3 CDH23 SLC17A5 NLGN3 CREBBP SOX2 CD36 ATXN10 DDHD2 LMNB1 VDR ATN1 CTLA4 HSPG2 NPHP1 SLC26A4 OFD1 SERPINE1 COQ2 ERCC5 DDHD1 MAP1B MYO7A EDN1 ALG13 GH1 RPS6KA3 EPM2A FOXP1 IFT140 IDS SNCB PEX26 DYNC1H1 LIMK1 XK GDAP1 SUMF1 VLDLR POMK H19 TSC1 MARS2 PTH PTPN22 INSR DCTN1 HGSNAT PRKAR1A GGT1 SMARCB1 MMP2 F5 SOX3 GNRH1 LTA COQ8A RUNX2 PEX21 |
| DisGeNet  AD (303) | APP APOE PSEN1 SORL1 BCL2 BDNF ACE GSK3B IL1B INSR LEP PLAU IGF2 NPY IGF1R INS BAX CLU PSEN2 PICALM ABCA7 CD2AP TREM2 CR1 VSNL1 PCDH11X MS4A4A A2M ACHE BIN1 BCHE CALM1 CASP3 CD33 CHRNA7 CST3 CYP2D6 DHCR24 DPYSL2 EPHA1 ESR1 HFE HMOX1 IDE IGF1 IL6 MAPT MPO MTHFR NOS3 PPARG PRNP RELN TNF VEGFA CYP46A1 NCSTN BACE1 MIR146A BLMH MAOB SOD2 TF TFAM APBB2 CHRNB2 SLC30A6 EIF2S1 ATP5F1A CRH ENO1 F2 IGF2R PAXIP1 ARC HLA-DRB5 TPI1 SLC30A4 ABI3 AGER AMFR CHAT TPP1 LRP1 MME NGF NGFR PIN1 PLCG2 PYY PTGS2 S100B SLC2A4 SOD1 GAPDHS NFE2L2 NOS2 MAP2 NTRK2 IRS1 APLP2 PTGS1 PPARGC1A NGB CAV1 MAPK14 CTNNB1 CCR5 HSPB1 HSPD1 MFN2 CIB1 ATP7A GSR HSF1 IKBKB INS-IGF2 MT2A SERPINF1 GAB2 TOMM40 PLCB1 SQSTM1 FERMT2 EXOC3L2 MS4A4E NFIC CELF1 ZCWPW1 FRMD4A MS4A6A PTK2B IL6R INPP5D TRIP4 BCL3 NR3C2 SUCLG2 SPON1 CELF2 MTHFD1L DCHS2 CASS4 EXOC4 MEGF10 PLXNA4 SLC24A4 GLIS3 SERPINA3 ABCA1 ADAM10 AP2A2 ALOX5 APBB1 APOA1 DST RUNX1T1 CDK5 CDR1 CETP CHRNA4 CLPTM1 ACKR2 CP CRP CSF2 CTNNA2 CTSB CTSD DMXL1 DLG4 RCAN1 DYRK1A ENO2 ESR2 MTOR GABRG3 GAPDH GATA1 GFAP GRN GRIN2B HSD17B10 HTR2A IL1A IL10 KCNN2 LAMC2 RPSA LDLR LTBP2 BCAM AFF1 MMP9 MOBP COX2 RNR2 NTF3 ABCB1 PON1 PTPA MAPK1 PTPRA PTPRG NECTIN2 MOK CCL2 ST6GAL1 SLC1A2 SLC6A4 SNCA SST SYP TGFB1 TP53 TTR UBB UCHL1 AGPS CRADD SUCLA2 CDK5R1 CACNA1G CCRL2 SLC16A7 LRAT ITM2B SLC4A8 ST18 MVP DNM1L GPC6 FARP1 MYO16 ZNF292 FBXL7 PRRC2C CLEC16A WWC1 ACSL6 SIRT1 NCS1 TARDBP SMUG1 BACE2 TRPC4AP PDE7B TNRC6A BZW2 PARVB IL19 UBQLN1 RMDN1 RAPGEF6 BCAS3 CDKAL1 RMDN3 STK32B SYBU EDEM2 FMN2 CDC42SE2 ARHGAP20 SH3RF1 VAT1L CSMD1 PPP1R3B ANKRD55 CLMN SP6 SPPL2A FNIP1 PALM2 OSBPL6 LRRK2 ANO4 NDUFAF6 RMDN2 NKAIN2 BMPER PLPP4 C9orf72 CCDC83 STH CALHM1 PPP1R37 NKPD1 EPHA1-AS1 LUZP2 TGM6 MCIDAS SIMC1 SCIMP LINC01567 PALM2-AKAP2 LINC01184 MEIKIN SLC8A1-AS1 ARL17B TSPOAP1-AS1 LINC01725 MEF2C-AS1 LINC00972 |
| MCI (5) | APP BDNF CSF2 LAMC2 APOE |
| OMIM  AD (523) | AAOPD AAT6 ABBP2 ABC1 ABCA1 ABCA4 ABCB1 ABCB4 ABCG8 ABCR AC133 ACTA2 ACTSA AD10 AD11 AD12 AD13 AD14 AD15 AD17 AD4 AD6 AD7 AD7CNTP ADAM17 ADH1C ADH3 ADMD ADMIO2 ADSD AGL AILJK AIS1 AIS2 AIS3 AIS4 AIS5 AITD1 AITD2 AITD3 AITD4 ALPS5 ALS11 ALS14 AMOXAD ANDD AOS5 AOVD1 APBD APC APG16L ARC41 ARHR2 ARMD2 ARPC1B ARPKD ASD2 ATG16L1 ATGL ATP1A1 ATP2C1 BCATE2 BCKDHB BCPM BDPLT10 BDPLT5 BMPR2 BSF2 BTOP BTPS2 C1orf106 C3orf9 CAAHD CALHM1 CALLA CAV3 CCDD CCHS CD10 CD133 CD36 CELIAC1 CELIAC10 CELIAC11 CELIAC12 CELIAC2 CELIAC3 CELIAC6 CELIAC7 CELIAC8 CELIAC9 CESD CHCHD2 CHDS2 CHDS5 CHDS7 CHDS8 CHDS9 CHN1 CHN2 CLCs CLP46 CMD1A CMD1V CMH6 CMRD CMT1B CMT1F CMT1G CMT2A CMT2A1 CMT2A2A CMT2A2B CMT2B CMT2D CMT2DD CMT2E CMT2EE CMT2F CMT2H CMT2K CMT2P CMT2Q CMT2R CMT2T CMT2W CMT2Y CMT4A CMT4B2 CMT4D CMT4K CMTD1F CMTDIA CMTDIC CMTDID CMTDIG CMTRIA CMTRIC COLED COPA COPD CORD12 CORD3 CRB2 CSIF CTLA4 CX3CR1 D10S105E DBP2 DBT DDD4 DDX16 DESMD DFNB57 DHTKD1 DHX16 DJ1 DJ9 DJC6 DMRV DNAJB11 DNAJC6 DSMA4 DSS DZIP1L DZIP2 E1B ECE1 ECM1 EGFR EGR2 EIF4G EIF4G1 ELOVL4 EMD2 ENPP1 ESA FAM26C FARSLB FCYT FFM FIG4 FOXD3 FPC FPLD2 FRPHE FRSB FSGS9 FTDALS3 GARS GARS1 GATA4 GBA GBD2 GBD3 GBD4 GBE1 GDA GDAP1 GDE GDNF GIGYF2 GLE1 GLE1L GNB4 GPR13 GS GSD10 GSD11 GSD15 GSD4 GVHDS GYG1 HARS HARS1 HD HDL3 HDL4 HDLCQTL13 HEDJ HEXB HFE HFE1 HGF HGPS HHD HLA-DPB1 HLA-DQA1 HLA-DQB1  HLA-HHLN2 HMN2B HMN5 HMSN6A HMSNL HNRPA2B1 HOMGSMR2 HOX4D HOXD10 HPALP1 HSCR1 HSCR3 HSCR5 HSCR6 HSCR9 HSF HSP27 HSPB1 HTRA2 HTT IBD10 IBD11 IBD12 IBD13 IBD14 IBD15 IBD16 IBD17 IBD18 IBD19 IBD20 IBD23 IBD29 IBD3 IBD5 IBD7 IBD9 IBMPFD1 IBMPFD2 ICP3 IDDM12 IFI1 IFI41 IFI75 IFNB2 IL10 IL23R IL6 IMD48 INAVA IRF5 IRGM IRS1 ISQMR IT15 JP1 JPH1 KIAA0214 KIAA0274 KIAA0473 KIAA0517 KIAA0642 KIAA0720 KIAA1441 KIAA1630 KIAA1985 KIF1B KROX20 KTELC1 LCCS LCCS1 LDH1 LDHA LGMDR21 LIPA LMN1 LMNA LOMARS LPA LQT9 LRG47 LRSAM1 M6S1 MCDR2 MCKD1 MDM MDR1 MDR3 MELIOS MEN2A MFN2 MGCA8 MME MNMN MPDT MPV17 MPZ MSUDMV MTDPS6 MTHFR MTMR13 MUC1 MVCD5 MVCD7 MYMY MYMY1 MYMY3 MYMY5 NACP NADGP NBLST1 NBLST2 NBLST6 NBPHOX NCF1 NCF2 NDGOA NDRG1 NEFL NEP NIID NISBD1 NISBD2 NMOAS NOS3 NOTCH1 NOTCH2NLC NPD NPPS OCIF OMI OPG P62 PAHX PAOD1 PARK1 PARK10 PARK11 PARK13 PARK16 PARK18 PARK19 PARK2 PARK21 PARK22 PARK3 PARK4 PARK5 PARK6 PARK7 PCA1 PCLD2 PDB3 PDB4 PDB5 PDB6 PDCD1 PDE11A PDE11A1 PDE11A2 PDE11A3 PDE8B PDJ PDNP1 PDZD7 PGAM2 PGAMM PGY1 PGY3 PHOX2B PHYH PINK1 PKD2 PKD4 PKD5 PKD6 PKHD1 PLAU PLEKHG5 PLTEID PMP2 PMX2B PNPLA2 POGLUT1 PON PON1 PON2 POVD1 PP2CM PPH1 PPM1K PPNAD2 PPNAD3 PRKAG2 PRKN PROM1 PROML1 PRP8 PRSS25 PSAP PSEN2 PSN PTMP PUM PYL QPD RAB7 RET RHCE RHNA RIFLE RILDBC RMD1 RMD2 RP19 RP41 RP47 RUMI SAC3 SAG SAP1 SAR1B SARA2 SBF2 SCA17 SCA34 SCA43 SEC63 SFRP4 SH3TC2 SIASD SLC17A5 SLC25A16 SLD SLEB1 SLEB10 SLEB2 SLURP1 SMAD1 SMPD1 SNCA SP110 SPG79 SQSTM1 SRK STGD1 STGD2 STGD3 STGD4 STM2 STSL1 SURF1 TACE TACHD TAL TAN1 TBP TDH3 TFQTL2 TG TGD TIL3 TLR5 TNFRSF11B TNNI3K TOF TRIM2 TTS2 TYRRS UCHL1 URBWD URK USH3B V28 VAMAS2 VAMAS3 VAMAS4 VAMAS5 VAMAS6 VCP VMCKD VODI VSD1 WPWS YARS YARS1 YRS YTS YVS ZAP70 ZFAT1 ZNF406 ZNF687 |
| MCI (85) | 1C7 AMMECR1 AR1 ARHI1 ARHI2 ARVD11 ATPSK2 BCYM4 BFIS5 BSND C11orf9 CAVIPMR CD337 CGF1 CIAT COB1 COL2A1 CUGS DDVIBA DDX30 DHPS DHX30 DNAJC12 DSC2 DSC3 EIEE13 EMARDD EMC1 FOXP1 GB5 GNB5 HOXA2 HPANBH4 IDDCA JDP1 JMJD3 KDM6B KIAA0090 KIAA0346 KIAA0890 KIAA0954 KIAA1780 KIAA1985 LADCI LBR MALS MARK3 MCOHI MEGF10 MFHIEN MMERV MNMN MSUDMV MYOCL2 MYRF NCR3 NEDCFSA NEDMIAL NEDSSWI NKP30 NR0B2 PAH PAPSS2 PHA PHASK PKU1 PP2CM PPM1K PTMP QRF1 RETCOR SCN8A SCZD3 SH3TC2 SHP SLI1 SLI2 SLI3 SLI4 SLI5 SPBP TCF20 TM4SF20 VIPB YAP1 |
| AD-MCI Common Target  (236) | ABCA1 ABCA7 ACE ACHE ADNP ADORA2A AGPS AKT1 ALB APOA1 APOB APOC1 APOE APP ARSA ATN1 ATP13A2 ATXN1 ATXN2 ATXN3 BACE1 BCHE BCL2 BDNF C4A C9orf72 CACNA1A CACNA1C CASP1 CASP3 CAT CCL2 CD33 CD36 CD40 CD40LG CDR1 CFH CHAT CHMP2B CHRNA4 CHRNA7 CLU COMT CP CRH CRP CRYAB CSF1R CSF2 CST3 CTLA4 CTNNB1 CTSB CTSD CYCS CYP2D6 CYP46A1 DBH DCTN1 DNM1L DNMT1 DRD2 DRD3 DRD4 DYNC1H1 EDN1 EGF EPHA1 EPM2A ESR1 ESR2 F2 FAS FGFR1 FMR1 FUS GAL GBA GDAP1 GDNF GFAP GHRL GJA1 GJB1 GRIA3 GRIN1 GRIN2A GRIN2B GRN GSK3B  HFE HLA-B HLA-DQB1 HLA-DRB1 HMOX1 HSPD1 HSPG2 HTR1A HTR2A HTT ICAM1 IFNG IGF1 IGF2 IL10 IL1B IL6 INS INSR ITPR1 JAG1 JPH3 KIAA1985 KRT14 LAMC2 LDLR LEP LMNA LPL LRP2 LRRK2 LTA MAOA MAP1B MAPK1 MAPT MCIDAS MECP2 MEGF10 MFN2 MIR132 MME MMP2 MMP9 MNMN MPO MPZ MS4A6A MSUDMV MT-ATP6 MT-CO1 MT-CO2 MTHFR MT-ND1 MTOR MTR NDUFS3 NDUFS4 NEFL NFE2L2 NGF NOS2 NOS3 NOTCH1 NOTCH3 NPC1 NTF3 NTRK1 NTRK2 PARK7 PDYN PICALM PIN1 PINK1 PLA2G6 PLP1 POLG POMC PON1 PP2CM PPARG PPM1K PRKN PRNP PRODH PSAP PSEN1 PSEN2 PTEN PTMP RAB5A RAB7A RELN RET RUNX1T1 RYR1 SEMA3A SERPINE1 SH3TC2 SIRT1 SLC17A5 SLC18A2 SLC1A2 SLC1A3 SLC2A1 SLC6A3 SLC6A4 SMUG1 SNAP25 SNCA SNCB SOD1 SORL1 SOX10 SPAST SPG21 SQSTM1 STAT3 SYNJ1 SYP TARDBP TBK1 TBP TGFB1 TH TLR2 TLR4 TMEM106B TNF TNFRSF1A TNFRSF1B TOMM40 TP53 TPP1 TREM2 TSPO TTR UBQLN1 UCHL1 VCP VDR VEGFA VLDLR VWF WWC1 |

TABLE 4: The characteristics of GO enrichment analysis.

| Biological Processes (BP)  GO:0042493~response to drug  GO:0045944~positive regulation of transcription from RNA polymerase II promoter  GO:0008284~positive regulation of cell proliferation  GO:0043066~negative regulation of apoptotic process  GO:0010628~positive regulation of gene expression  GO:0045893~positive regulation of transcription, DNA-templated  GO:0007568~aging  GO:0006954~inflammatory response  GO:0051091~positive regulation of sequence-specific DNA binding transcription factor activity  GO:0001934~positive regulation of protein phosphorylation | PValue  5.72311E-10  0.00206387  2.23307E-06  1.96661E-05  6.8801E-07  0.000717324  1.28736E-07  0.000372626  1.86468E-06  8.5044E-06 |
| --- | --- |
| Cellular Components (CC)  GO:0005615~extracellular space  GO:0005576~extracellular region  GO:0005886~plasma membrane  GO:0045121~membrane raft  GO:0030424~axon  GO:0009986~cell surface  GO:0005739~mitochondrion  GO:0005887~integral component of plasma membrane  GO:0070062~extracellular exosome  GO:0005829~cytosol | PValue  3.91847E-16  2.71118E-12  8.85509E-08  1.3331E-07  4.34792E-05  0.000158526  0.000352764  0.000613841  0.000651171  0.004424818 |
| Molecular Function (MF)  GO:0005515~protein binding  GO:0042802~identical protein binding  GO:0019899~enzyme binding  GO:0005125~cytokine activity  GO:0005102~receptor binding  GO:0008083~growth factor activity  GO:0002020~protease binding  GO:0008144~drug binding  GO:0051721~protein phosphatase 2A binding  GO:0001540~beta-amyloid binding | PValue  0.000616873  1.57133E-07  9.42687E-08  0.000312959  0.028028156  0.002877499  0.004051044  0.02377169  0.020533419  0.040931288 |

TABLE 5: The characteristics of KEGG enrichment analysis.

| Pathway | Count | PValue | Target |
| --- | --- | --- | --- |
| Pathways in cancer | 16 | 3.32903E-08 | IL6 MMP9 PPARG TP53 RUNX1T1 PTEN MMP2 TGFB1 AKT1 MAPK1 CASP3 GSK3B BCL2 VEGFA NOS2 EGF |
| HIF-1 signaling pathway | 12 | 3.21932E-11 | AKT1 MAPK1 IL6 HMOX1 BCL2 VEGFA SERPINE1 IFNG NOS3 NOS2 EGF INSR |
| Tuberculosis | 12 | 2.37081E-08 | AKT1 MAPK1 CASP3 IL6 TNF BCL2 IFNG IL1B CTSD NOS2 TGFB1 IL10 |
| Chagas disease (American trypanosomiasis) | 11 | 1.63282E-09 | AKT1 MAPK1 IL6 TNF CCL2 SERPINE1 IFNG IL1B NOS2 TGFB1 IL10 |
| Proteoglycans in cancer | 11 | 8.60302E-07 | AKT1 MAPK1 CASP3 TNF MMP9 VEGFA TP53 ESR1 IGF2 MMP2 TGFB1 |
| PI3K-Akt signaling pathway | 11 | 0.000104693 | AKT1 MAPK1 IL6 GSK3B BCL2 VEGFA TP53 NOS3 EGF PTEN INSR |
| Toxoplasmosis | 10 | 4.89924E-08 | AKT1 MAPK1 CASP3 TNF CD40LG BCL2 IFNG NOS2 TGFB1 IL10 |
| Hepatitis B | 10 | 5.33675E-07 | AKT1 MAPK1 CASP3 IL6 TNF BCL2 MMP9 TP53 PTEN TGFB1 |
| Malaria | 9 | 1.11041E-09 | ICAM1 IL6 TNF CCL2 CD40LG IFNG IL1B TGFB1 IL10 |
| TNF signaling pathway | 9 | 5.93221E-07 | AKT1 ICAM1 MAPK1 CASP3 IL6 TNF CCL2 MMP9 IL1 |
| Influenza A | 9 | 2.26905E-05 | AKT1 ICAM1 MAPK1 IL6 TNF CCL2 GSK3B IFNG IL1B |
| MAPK signaling pathway | 9 | 0.00031525 | AKT1 MAPK1 CASP3 TNF MAPT TP53 IL1B EGF TGFB1 |
| Rheumatoid arthritis | 8 | 2.12004E-06 | ICAM1 IL6 TNF CCL2 VEGFA IFNG IL1B TGFB1 |
| Amoebiasis | 8 | 7.4041E-06 | CASP3 IL6 TNF IFNG IL1B NOS2 TGFB1 IL10 |
| Sphingolipid signaling pathway | 8 | 1.67912E-05 | AKT1 MAPK1 TNF BCL2 TP53 CTSD NOS3 PTEN |
| FoxO signaling pathway | 8 | 3.44187E-05 | AKT1 MAPK1 IL6 EGF PTEN INSR TGFB1 IL10 |
| Non-alcoholic fatty liver disease (NAFLD) | 8 | 7.39534E-05 | AKT1 CASP3 IL6 TNF GSK3B IL1B INSR TGFB1 |
| Alzheimer's disease | 8 | 0.000144778 | MAPK1 APP CASP3 TNF MAPT GSK3B BACE1 IL1B |
| Cytokine-cytokine receptor interaction | 8 | 0.001343541 | IL6 TNF CCL2 CD40LG IFNG IL1B TGFB1 IL10 |
| Colorectal cancer | 7 | 3.82777E-06 | AKT1 MAPK1 CASP3 GSK3B BCL2 TP53 TGFB1 |
